# Antibacterial and Cytotoxic Phenolic Polyketides from Two Marine-Derived Fungal Strains of *Aspergillus unguis*

**DOI:** 10.3390/ph15010074

**Published:** 2022-01-06

**Authors:** Cao Van Anh, Joo-Hee Kwon, Jong Soon Kang, Hwa-Sun Lee, Chang-Su Heo, Hee Jae Shin

**Affiliations:** 1 Marine Natural Products Chemistry Laboratory, Korea Institute of Ocean Science and Technology, 385 Haeyang-ro, Yeongdo-gu, Busan 49111, Korea; caovananh@kiost.ac.kr (C.V.A.); hwasunlee@kiost.ac.kr (H.-S.L.); science30@kiost.ac.kr (C.-S.H.); 2 Department of Marine Biotechnology, University of Science and Technology (UST), 217 Gajungro, Yuseong-gu, Daejeon 34113, Korea; 3 Laboratory Animal Resource Center, Korea Research Institute of Bioscience and Biotechnology, 30 Yeongudanjiro, Cheongju 28116, Korea; juhee@kribb.re.kr (J.-H.K.); kanjon@kribb.re.kr (J.S.K.)

**Keywords:** *Aspergillus unguis*, marine-derived fungus, phenolic polyketides, antibacterial, cytotoxicity

## Abstract

A chemical investigation on the EtOAc extracts from two marine-derived fungal strains of *Aspergillus unguis* resulted in the isolation of three previously undescribed phenolic polyketides including unguidepside C (**1**), aspersidone B (**3**), and agonodepside C (**12**), and their 14 known congeners. The structures of the new compounds were determined based on detailed analysis and comparison of their spectroscopic data with literature values, as well as Snatzke’s method. The new compounds (**1**, **3**, and **12**) displayed a significant anti-Gram-positive bacterial activity, with MIC values ranging from 5.3 to 22.1 µM. Additionally, the isolated compounds (**1**–**11** and **13**–**16**) were evaluated for their cytotoxicity against a panel of tumor cell lines. Most of them (except for **9**) displayed cytotoxicity against all the tested cell lines, with IC_50_ values ranging from 2.5 to 46.9 µM.

## 1. Introduction

Infectious diseases are a major health problem worldwide, killing more than nine million people annually, and they remain one of the most urgent global health challenges facing humanity [1]. Particularly, the rapid growth and global expansion of multi- and pan-resistant pathogens that are not treatable with presently available antimicrobial drugs have resulted in fewer treatment options for patients and an associated increase in mortality and morbidity [2]. After the golden era (1950s–1970s), the antibiotic pipeline began to dry up, and the discovery of new antibiotics has significantly diminished [2]. Therefore, there is a continuous demand for the discovery of new antimicrobial drugs.

In the last few years, natural products (NPs) isolated from marine-derived fungi have received great interest, as many of them are structurally unique and possess interesting pharmacological and biological properties. Among the marine-derived fungal genera, *Aspergillus* and *Penicillium* are the two largest suppliers of new fungal NPs and a significant portion of the isolated compounds showed antimicrobial or cytotoxic activity [3].

*Aspergillus unguis* is well known as a producer of numerous phenolic polyketides, which showed various pharmacological activities such as antibacterial, antifungal, cytotoxic, and antioxidant activities [4,5,6,7,8]. As part of our ongoing studies to discover new marine NPs with biological activities, the crude extracts from two strains of *A. unguis* IV17-109 and 158SC-067 showed good antibacterial activity and have been chemically investigated. In a previous study, we reported a new meroterpene, grifolin B, isolated from *A. unguis* 158SC-067 [9]. Further study on the EtOAc extracts of two strains resulted in the isolation of three previously undescribed phenolic polyketides (**1**, **3**, and **12**), and their 14 known congeners. In this paper, we describe the isolation, structure determination, and their anti-Gram-positive bacterial and cytotoxic activities.

## 2. Results and Discussion

**Figure 1:**
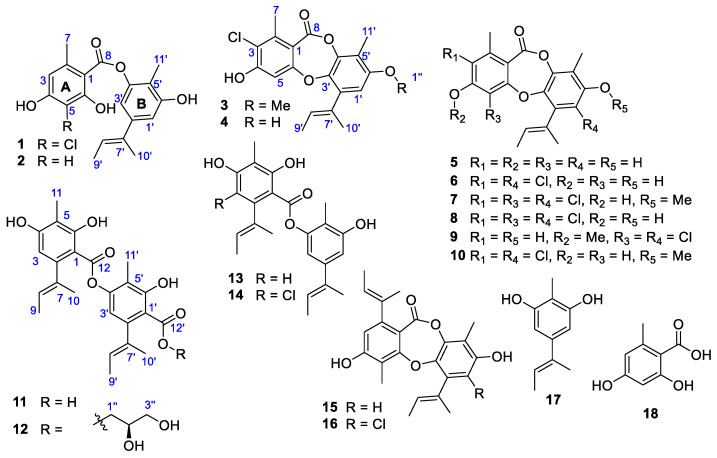
Structures of **1**–**17** isolated from two strains of *A. unguis* and orsellinic acid (**18**).

It has been recognized that *A. unguis* is a producer of phenolic polyketides (depsides and depsidones of nidulin derivatives) using orsellinic acid (**18**), and aspergillusphenol A (**17**) as building blocks. Some compounds, such as nidulin (**7**), nornidulin (**8**), unguinol (**5**), and agonodepside A (**13**) were repeatedly isolated from different strains of *A. unguis* as major secondary metabolites (SMs) [5,6,7]. During our study on NPs from marine-derived fungi, we isolated two fungal strains IV17-109 and 158SC-067, which were identified as *Aspergillus unguis* by ITS gene sequencing. The strains were cultivated in Bennett’s broth medium at 28 °C for two weeks, to investigate SMs. Consequently, we found that the two fungal strains produced many nidulin-related derivatives (Figure 1 and Figure S1), and agonodepside A (**13**) was isolated from both of the strains as one of the major SMs. Further study on the EtOAc extracts of the strains led us to the identification of three minor unreported congeners, and their chemical structures are characterized below.

**Figure 2:**
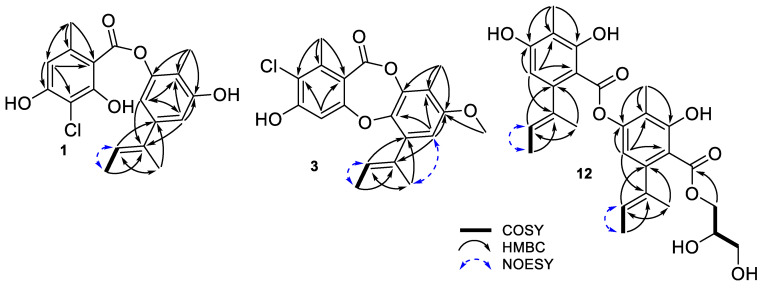
Key 2D NMR correlations of **1**, **3**, and **12**.

Compound **1** was obtained as a colorless solid. The observed HRESIMS peaks at *m/z* 385.0819 and 387.0796 [M + Na]^+^ with a ratio of 3:1 determined the molecular formula of **1** as C_19_H_19_ClO_5_ (calculated for C_19_H_19_ClO_5_Na, 385.0819, Figure S3), requiring 10 indices of hydrogen deficiency and containing one chlorine atom. The IR spectrum showed double-bond and hydroxy absorption bands at 1621 and 3417 cm^−1^, respectively. The UV spectrum showed absorption bands at 220 and 269 nm, indicating the presence of a benzene chromophore. The ^1^H NMR spectrum of **1** revealed eight signals assignable to two meta-coupling aromatic protons at *δ*_H_ 6.78 (d, *J* = 1.6, H-1′) and 6.62 (d, *J* = 1.6, H-3′); an aromatic singlet proton at *δ*_H_ 6.46 (s, H-3); an olefinic proton at *δ*_H_ 5.86 (m, H-8′); four methyl groups at *δ*_H_ 2.61 (s, H_3_-7), 2.00 (s, H_3_-11′), 1.98 (brs, H_3_-10′), and 1.77 (dd, *J* = 6.9, 0.7, H_3_-9′) (Table 1, Figure S4). The ^13^C NMR spectrum of **1**, with the help of the HSQC NMR spectrum, revealed nineteen signals of a carboxyl carbon at *δ*_C_ 171.4; ten non-protonated sp^2^ carbons at *δ*_C_ 161.9, 159.9, 157.5, 150.8, 144.2, 142.3, 135.9, 116.6, 107.2, and 106.1; four protonated sp^2^ carbons at *δ*_C_ 123.0, 112.4, 111.2, and 110.9; four methyl groups at *δ*_C_ 9.5–24.4 (Table 1, Figure S5). A carboxyl and 14 sp^2^ carbons accounting for eight out of ten indices of hydrogen deficiency indicated that **1** possesses a bicyclic skeleton (depside structure). The ^1^H and ^13^C NMR spectroscopic data of **1** quite resembled those of **2** (Figures S2, S25 and S26) except for the absence of an aromatic proton (H-5), and an increase in the molecular weight of 34 Da in **1**, compared with that in **2**, indicating **1** was a mono-chlorine-substituted analog of **2**. Further detailed analysis of 2D NMR data of **1** (Figures S6–S9) suggested the chlorine atom was substituted at the C-5 position of the ring A (Figure 2), and the structure of **1** was determined as 5-chlorodecarboxyunguidepside A and named unguidepside C.

Compound **3** was purified as a colorless solid. The HRESIMS peaks [M + Na]^+^ of **3** were observed at *m*/*z* 397.0818 and 399.0795 with a ratio of 3: 1, determining the molecular formula of **3** as C_20_H_19_ClO_5_ (calculated for C_20_H_19_ClO_5_Na, 397.0819, Figure S10), requiring 11 degrees of unsaturation and containing one chlorine atom. The ^1^H NMR spectrum of **3** (Table 1, Figure S11) showed the characteristic signals of four methyl groups at *δ*_H_ 2.44 (3 H, s, H_3_-7), 2.14 (3 H, s, H_3_-11′), 2.09 (3 H, bs, H_3_-10′), and 1.86 (3 H, d, *J* = 6.6, H_3_-9′); a methoxy group at *δ*_H_ 3.79 (3 H, s, H_3_-1′’); an olefinic proton at *δ*_H_ 5.60 (1 H, m, H-8′); two singlet aromatic protons at *δ*_H_ 6.53 (s, H-1′) and 6.53 (s, H-5). The ^13^C NMR spectrum showed twenty resonances, which were identified with the aid of the HSQC spectrum (Figures S12 and S13) as four methyls at *δ*_C_ 9.2–18.4; a methoxy at *δ*_C_ 56.5; three hydrogenated sp^2^ carbons at *δ*_C_ 106.3, 108.3, and 127.0; eleven quaternary sp^2^ carbons at *δ*_C_ 114.9, 118.1, 120.9, 134.3, 137.2, 142.7, 143.2, 144.5, 156.3, 159.1, and 162.8; a carbonyl group at *δ*_C_ 164.7. A carbonyl group and seven pairs of sp^2^ carbons accounting for eight out of eleven degrees of unsaturation indicated **3** possesses a three-cyclic backbone (depsidone structure). The ^1^H and ^13^C NMR spectroscopic data of **3** were quite similar to those of 3-chlorounguinol (**4**) (Figures S27 and S28), except for the presence of one more methoxy group (*δ*_C_ 56.5, *δ*_H_ 3.79), as well as an increase in the molecular weight of 14 Da in **3**, compared with that in **4**, indicating **3** was a new methylated derivative of **4**. Furthermore, the connection of the methoxy group to C-6′ was confirmed by the HMBC correlation from H_3_-1″ to C-6′ (3.79 to 156.3, Figure S15). Therefore, **3** was determined as 6′-methyl-3-chlorounguinol and named aspersidone B.

**Figure 3:**
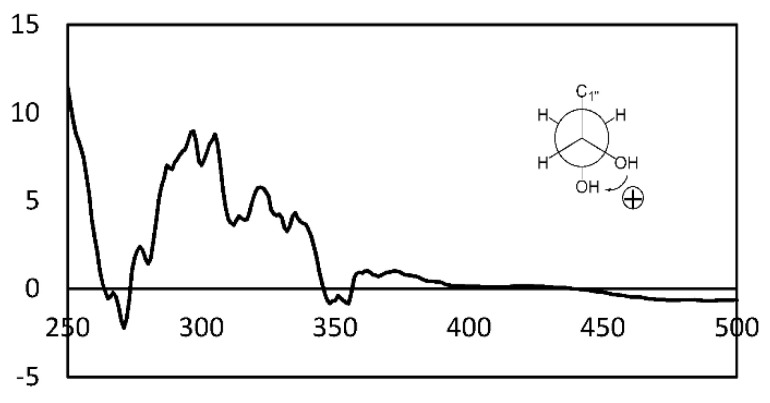
ICD spectrum of **12**.

Compound **12** was isolated as a white amorphous powder. The molecular formula of **12** was determined as C_27_H_32_O_9_ on the basis of HRESIMS data (*m*/*z* 523.1943 [M + Na]^+^, calculated for C_27_H_32_O_9_Na, 523.1944, Figure S17), requiring 12 degrees of unsaturation. The ^1^H and ^13^C NMR spectroscopic data of **12** (Table 2, Figures S18 and S19) were quite similar to those of agonodepside B (**11**), (Figures S29 and S30), except for the presence of two more oxygenated methylenes at *δ*_C_ 67.5 (C-1″) and 64.2 (C-3″), and one more oxygenated methine at *δ*_C_ 70.9 (C-2″). Further, this partial structure was determined as a glycerol moiety by the continuous ^1^H-^1^H COSY correlations from H_2_-1″ to H_2_-3″ (4.37 to 3.58, Figure S21). The glycerol moiety connected to the aglycone part via an ester bond from C-12′ to C-1″ was confirmed by the HMBC correlation from H-1″a,b (*δ*_H_ 3.58 and 3.62) to C-12′ (*δ*_C_ 171.9, Figure S22). The absolute configuration of 1,2-diol in the glycerol moiety was determined by the dimolybdenum-induced CD (ICD) method following Snatzke’s rule. The positive Cotton effects observed at 305 nm (band IV) and 375 nm (band II) in the ICD spectrum of **12** suggested the absolute configuration of C-2″ as 2″*R* (Figure 3 and Figure S24). Thus, the structure of **12** was determined as agonodepside B glyceride and named agonodepside C.

The structures of the known compounds were determined as decarboxyunguidepside A (**2**) [7,10], 2-chlorounguinol (**4**) [6], unguinol (**5**) [11,12], 3,1′-dichlorounguinol (**6**) [13], nidulin (**7**) [11], nornidulin (**8**) [11], aspergillusidone B (**9**) [14], aspersidone (**10**) [11], agonodepside B (**11**) [5], agonodepside A (**13**) [5], guisinol (**14**) [4], folipastatin (**15**) [11], emeguisin A (**16**) [11], and aspergillusphenol A (**17**) [8] by comparing their spectroscopic data with those reported in the literature.

Since the known depsides and depsidones isolated from *Aspergillus unguis* showed potent antimicrobial or cytotoxic activity. The new compounds (**1**, **3**, and **12**) were evaluated for their antimicrobial activity against three Gram-negative bacteria: *Klebsiella pneumonia* (KCTC 2690), *Salmonella typhimurium* (KCTC 2515), and *Escherichia coli* (KCTC 2441); three Gram-positive bacteria were also analyzed—namely, *Staphylococcus aureus* (KCTC 1927), *Micrococcus luteus* (KCTC 1915), and *Bacillus subtilis* (KCTC 1021). Compounds **1**, **3**, and **12** showed antimicrobial activity against all the tested Gram-positive bacteria (KCTC 1021, 1915, and 1927), with MIC values ranging from 5.3 to 22.1 μM (Table 3). However, the tested compounds did not inhibit the growth of Gram-negative bacteria at the concentration of 128.0 μg/mL.

In addition, the isolated compounds (**1**–**11** and **13**–**16**) were also evaluated for their cytotoxicity against a panel of cancer cell lines, including PC-3 (prostate), NCI-H23 (lung), HCT-15 (colon), NUGC-3 (stomach), ACHN (renal), and MDA-MB-231 (breast), which are the most common cancer types in Korea. Most of the tested compounds (except for **9**) showed cytotoxic activity against all the tested cell lines, with IC_50_ values ranging from 2.5 to 46.9 µM (Table 4). Considering cytotoxic activity results, it could be noteworthy that phenolic polyketides isolated from *A. unguis* showed cytotoxicity against cancer cell lines with a different tendency. The substitution and number of chlorine atoms did not significantly increase the cytotoxicity of these compounds. The presence of a free carboxylic acid (**11**) [5,7] resulted in a significant reduction in cytotoxic activity, and the free hydroxy group at C-4 (**9**) is essential for cytotoxicity [14]. 

## 3. Materials and Methods

### 3.1. General Experimental Procedures

HRESIMS spectra were acquired on Waters Synapt HDMS LC/MS mass spectrometer (Waters Corporation, Milford, MA, USA). Optical rotations were recorded using a Rudolph Research Analytical Autopol III polarimeter (Rudolph Research Analytical, Hackettstown, NJ, USA). In addition, 1D and 2D NMR spectra were measured on a Bruker 600 MHz spectrometer (Bruker BioSpin GmbH, Rheinstetten, Germany). IR spectra were obtained on a JASCO FT/IR-4100 spectrophotometer (JASCO Corporation, Tokyo, Japan). HPLC was performed with PrimeLine Binary pump (Analytical Scientific Instruments, Inc., El Sobrante, CA, USA) and RI-101 (Shoko Scientific Co., Ltd., Yokohama, Japan). Semipreparative HPLC was carried out with an ODS column (YMC-Pack-ODS-A, 250 × 10 mm i.d, 5 μM). Analytical HPLC was conducted using an ODS column (YMC-Pack-ODS-A, 250 × 4.6 mm i.d, 5 μM).

### 3.2. Fungal Strains, Cultivation, and Isolation of Secondary Metabolites

#### 3.2.1. Fungal Strain, Cultivation, and Isolation of **11**–**13** and **15** from *A. unguis* 158SC-067

*Aspergillus unguis* 158SC-067 was isolated from a seawater sample, as previously described [9]. The mycelium extract was separated into 10 fractions (1–10 m), as described earlier [9]. Fraction 8 m was subjected to an analytical HPLC (YMC-Pack ODS-A, 250 × 4.6 mm i.d., 5 µm, flow rate 0.9 mL/min) with an isocratic elution of 70% MeOH in H_2_O to obtain compounds **11** (3.0 mg, t_R_ = 10 min) and **15** (3.0 mg, t_R_ = 24 min). Compounds **12** (1.3 mg, t_R_ = 25 min) and **13** (3.0 mg t_R_ = 29 min) were purified from fraction 9 m by a semipreparative HPLC (YMC-Pack ODS-A, 250 × 10 mm i.d., 5 µm, flow rate 2.0 mL/min) with an isocratic elution of 80% MeOH in H_2_O.

#### 3.2.2. Fungal Strain, Cultivation, and Isolation of **1**–**10**, **14**, and **16**–**17** from *A. unguis* IV17-109

The strain IV17-109 was isolated from a deep-sea shrimp sample (*Rimicaris* sp.) collected using a TV grab on the ship R/V ISABU from the Indian Ocean (date: 31 July 2017, latitude: 019° 33.3935′ S, longitude: 065° 50.9136′ E, depth: 2632 m). The strain was identified as *Aspergillus unguis* on the basis of DNA amplification and ITS gene sequencing. The sequence data of IV17-109 was deposited at GenBank under accession number OL700797. Currently, the strain is stored at the Lab of Marine Microbial Natural Product, KIOST, with the name of *Aspergillus* sp. IV17-109, under the curatorship of Hee Jae Shin.

The strain was cultivated on a Petri dish containing Bennett’s agar medium (glucose 10.0 g/L, yeast extract 1.0 g/L, beef extract 1.0 g/L, tryptone 2.0 g/L, glycerol 5.0 g/L, sea salt 32 g/L, and agar 17 g/L) for 7 days. The actively growing mycelium was transferred aseptically into a 500 mL conical flask containing 300 mL of Bennett’s broth medium and incubated on a rotary shaker (140 rpm) at 28 °C for 5 days, to make the seed culture. An aliquot (0.1% *v*/*v*) from the seed culture was inoculated into forty 2 L flasks each containing 1 L of the broth medium and grown under the same conditions as described for the seed culture for 14 days and then harvested.

After cultivation, the cultures were filtered through cheesecloth to separate broth and mycelium. The mycelium was extracted with 3 × 2 L of EtOAc, and the broth was extracted twice with an equal volume of EtOAc. The EtOAc extracts were combined and evaporated under reduced pressure to yield a crude extract (4.0 g). The crude extract was further separated into 10 fractions (F1–F10) by vacuum liquid chromatography on an ODS column using a stepwise gradient elution, with 200 mL each of 10–90% MeOH in H_2_O and 100% MeOH. The F5 fraction was purified by an analytical HPLC (YMC-Pack-ODS-A, 250 × 4.6 mm i.d, 5 µm, flow rate 1.0 mL/min, UV detector) using a gradient elution with 10–30% ACN/H_2_O, 0–60 min to yield compound **17** (1.2 mg, *t*_R_ = 40 min). The F7 fraction was subjected to a semipreparative reversed-phase HPLC (YMC-Pack-ODS-A, 250 × 10 mm i.d, 5 µm, flow rate 2.0 mL/min, RI detector) using an isocratic elution with 60% MeOH/H_2_O to obtain compounds **5** (7.0 mg, *t*_R_ = 46 min) and **4** (10.0 mg, *t*_R_ = 87 min). The F8 fraction was purified by a semipreparative reversed-phase HPLC (YMC-Pack-ODS-A, 250 × 10 mm i.d, 5 µm, flow rate 2.0 mL/min, RI detector) using an isocratic elution with 70% MeOH/H_2_O to yield compounds **2** (1.3 mg, *t*_R_ = 39 min), **1** (1.2 mg, *t*_R_ = 45 min), **16** (1.5 mg, *t*_R_ = 62 min), **6** (6.0 mg *t*_R_ = 76 min), and **8** (5.0 mg, *t*_R_ = 82 min). Finally, compounds **14** (2.0 mg, *t*_R_ = 24 min), **3** (2.0 mg, *t*_R_ = 28 min), **9** (1.3 mg, *t*_R_ = 32 min), **10** (1.5 mg, *t*_R_ = 40 min), and **7** (5.0 mg, *t*_R_ = 56 min) were purified from the F9 fraction by a semipreparative reversed-phase HPLC (YMC-Pack-ODS-A, 250 × 10 mm i.d, 5 µm, flow rate 2.0 mL/min, RI detector) using an isocratic elution with 83% MeOH/H_2_O.

Unguidepside C (**1**): colorless solid; IR ν_max_ 3417, 2971, 1657, 1621, 1572, 1420, 1299, 1257, 1031 cm^−1^; UV(MeOH) λ_max_ (log ε) 269 (2.90), 220 (3.35) nm; HRESIMS *m*/*z* 385.0819 [M + Na]^+^ (calculated for C_19_H_19_ClO_5_Na, 385.0819), ^1^H NMR (CD_3_OD, 600 MHz) and ^13^C NMR (CD_3_OD, 150 MHz) see Table 1.

Aspersidone B (**3**): colorless solid; IR ν_max_ 2971, 1717, 1593, 1420, 1222, 1006 cm^−1^; UV(MeOH) λ_max_ (log *ε*) 209 (3.50) nm; HRESIMS *m*/*z* 397.0818 [M + Na]^+^ (calculated for C_20_H_19_ClO_5_Na, 397.0819), ^1^H NMR (CD_3_OD, 600 MHz) and ^13^C NMR (CD_3_OD, 150 MHz) see Table 1.

Agonodepside C (**12**): white powder; ${\left[\alpha \right]}_{D}^{25}$—10 (*c* 0.1, MeOH); IR ν_max_ 3385, 2967, 1731, 1649, 1458, 1261, 1031 cm^−1^; UV(MeOH) λ_max_ (log *ε*) 278 (3.05), 218 (3.40) nm; HRESIMS *m*/*z* 523.1943 [M + Na]^+^ (calculated for C_27_H_32_O_9_Na, 523.1944), ^1^H NMR (CD_3_OD, 600 MHz) and ^13^C NMR (CD_3_OD, 150 MHz); see Table 2.

### 3.3. Snatzke’s Method

At first, the ECD spectrum of **12** was measured to establish as a background spectrum, and then, the ICD spectrum of **12** was obtained according to the published methods [15,16].

### 3.4. Antibacterial Assay

The antimicrobial activity of compounds **1**, **3**, and **12** was evaluated by using a standard broth dilution assay [17]. Compounds **1**, **3**, and **12** were tested against three Gram-negative bacteria: *Klebsiella pneumonia* (KCTC 2690), *Salmonella typhimurium* (KCTC 2515), and *Escherichia coli* (KCTC 2441); three Gram-positive bacteria were also included—namely, *Staphylococcus aureus* (KCTC 1927), *Micrococcus luteus* (KCTC 1915), and *Bacillus subtilis* (KCTC 1021). A serial double dilution of each compound was prepared in 96-microtiter plates in the range of 0.5–256 μg/mL. An overnight culture broth of each strain was dispensed in sterilized 0.9% saline to an inoculum density of 5 × 10^5^ cfu by comparison with a MacFarland standard. The diluted culture broth (100 μL) was added to each dilution of the tested compounds (**1**, **3**, and **12**, (100 μL)), in the plate to yield final concentrations from 0.25 to 128 μg/mL. The plates were incubated for 24 h at 37 °C. The MIC value is the lowest concentration at which the microorganism did not demonstrate visible growth, as indicated by the presence of turbidity. Kanamycin was used as a positive control. All experiments were conducted twice to check reproducibility [17].

### 3.5. Sulforhodamine B (SRB) Assay for Cytotoxicity Test

Cytotoxicity by SRB assay was performed according to published procedures, using adriamycin as a positive control [18]. The test was conducted with two independent experiments using duplicate measurements (Figure S31).

## 4. Conclusions

In summary, 17 phenolic polyketides, including 3 new and 14 known compounds, were isolated from 2 fungal strains of *Aspergillus unguis* IV17-109 and 158SC-067. The structures of the new compounds were determined by spectroscopic (HRESIMS, 1D and 2D NMR), and Snatzke’s methods. The new compounds (**1**, **3**, and **12**) showed good antimicrobial activity. Additionally, cytotoxicity of phenolic polyketides from *A. unguis* against six cancer cell lines was systematically investigated for the first time. Most of the isolated compounds showed cytotoxicity against all tested cell lines, with IC_50_ values ranging from 2.5 to 46.9 µM. This study enriched the biochemical diversity of fungal secondary metabolites. The results revealed that marine-derived fungi could be a promising source of natural products, with various pharmacological activities, which can serve as leading structures for drug discovery.

## Figures and Tables

**Table 1:** ^1^H and ^13^C NMR data of **1** and **3** in CD_3_OD (600 MHz for ^1^H and 150 MHz for ^13^C).

Compound	1		3	
Position	*δ*_H,_ Mult (*J* in Hz)	*δ*_C_	*δ*_H,_ Mult (*J* in Hz)	*δ*_C_
1		106.1		114.9
2		142.3		142.7
3	6.47, s	112.4		120.9
4		159.9		162.8
5		107.2	6.53, s	106.3
6		161.9		159.1
7	2.61, s	24.4	2.44, s	18.4
8		171.4		164.7
1′	6.78, d (1.6)	110.9	6.53, s	108.3
2′		144.2		137.2
3′	6.62, d (1.6)	111.2		143.2
4′		150.8		144.5
5′		116.6		118.1
6′		157.5		156.3
7′		135.9		134.3
8′	5.86, m	123.0	5.60, m	127.0
9′	1.77, dd (0.7, 6.9)	14.3	1.86, d (6.6)	14.0
10′	1.98, brs	15.4	2.09, brs	17.9
11′	2.00, s	9.5	2.14, s	9.2
1″			3.79, s	56.5

**Table 2:** ^1^H and ^13^C NMR data of **12** in CD_3_OD (600 MHz for ^1^H and 150 MHz for ^13^C).

Position	*δ*_H,_ Mult (*J* in Hz)	*δ*_C_	Position	*δ*_H,_ Mult (*J* in Hz)	*δ*_C_
1		103.0	1′		111.2
2		149.6	2′		148.3
3	6.20, s	110.4	3′	6.37, s	115.4
4		162.4	4′		153.8
5		110.9	5′		118.4
6		163.8	6′		161.4
7		139.9	7′		139.0
8	5.35, m	121.9	8′	5.35, m	123.2
9	1.66, d (6.1)	13.8	9′	1.74, d (6.1)	14.0
10	1.93, s	18.8	10′	1.93, s	19.1
11	2.06, s	8.0	11′	2.00, s	9.4
12		170.6	12′		171.9
1″	4.30, dd (5.9, 11.3) 4.37, dd (4.9, 11.3)	67.5			
2″	3.90, dt (5.9, 10.8)	70.9			
3″	3.62, dd (5.2, 11.3) 3.58, dd (5.7, 11.2)	64.2			

**Table 3:** Antibacterial activities of **1**, **3**, and **12**.

MIC (µM)			
	*B. subtilis* KCTC 1021	*Micrococcus luteus* KCTC 1915	*Staphylococcus aureus* KCTC 1927
**1**	22.1	22.1	22.1
**3**	10.7	10.7	5.3
**12**	8.0	16.0	16.0
Kanamycin	1.0	0.5	1.0

**Table 4:** Growth inhibition (GI_50_, µM) of **1**–**11** and **13**–**16** against human cancer cell lines.

Compounds	ACHN	NCI-H23	PC-3	NUGC-3	MDA-MB-231	HCT-15
**1**	13.9	19.6	16.1	7.8	16.9	13.2
**2**	2.5	2.9	2.7	2.6	3.1	3.0
**3**	12.9	14.0	14.4	8.3	15.5	12.5
**4**	4.6	4.0	3.7	3.4	4.6	3.9
**5**	5.0	4.4	4.4	3.4	6.0	6.2
**6**	4.8	4.7	4.8	3.8	5.5	5.2
**7**	27.7	16.1	26.6	18.9	24.3	24.6
**8**	7.3	9.8	7.5	4.3	13.3	10.5
**9**	n.a.	n.a.	n.a.	n.a.	n.a.	n.a.
**10**	11.8	11.9	10.2	7.7	7.8	9.1
**11**	43.8	41.8	32.8	26.3	26.9	46.9
**12**	n.t.	n.t.	n.t.	n.t.	n.t.	n.t.
**13**	2.7	3.7	3.1	1.9	3.7	4.9
**14**	13.4	11.2	11.5	10.4	13.0	13.0
**15**	14.8	13.8	16.9	13.9	12.9	14.3
**16**	16.5	12.4	14.0	13.2	14.6	10.5
**17**	n.t.	n.t.	n.t.	n.t.	n.t.	n.t.
Adr.	0.15	0.12	0.15	0.15	0.16	0.15

Adr., Adriamycin as a positive control. GI_50_ values are the concentration corresponding to 50% growth inhibition. “n.a.” means not active at the concentration of 50 µM. “n.t.” means not tested due to limited amount of sample.

## Data Availability

The Data presented in the article and the supplementary materials are available.

## Supplementary Materials

The following supporting information can be downloaded at: https://www.mdpi.com/article/10.3390/ph15010074/s1, Figure S1. Structures of **1**–**17** isolated from *A. unguis* IV17-109 and 158SC-067 (detailed). Figure S2. Comparison of chemical shifts of the new compounds (**1**, **3**, and **12**) with those of their analogs. Figures S3–S23: HRESIMS data, ^1^H and ^13^C, HSQC, ^1^H-^1^H COSY, HMBC, NOESY NMR spectra of **1**, **3**, and **12**. Figure S24. Comparison of optical rotation signs between **12** and other glycerides of carboxylic acids. Figures S25–S30: ^1^H and ^13^C NMR spectra of **2**, **4**, and **11**. Figure S31. Results of the cytotoxicity test of compounds **1**–**11** and **13**–**16**.
