# Supplementary material for: Antibacterial and Cytotoxic Phenolic Polyketides from Two Marine-Derived Fungal Strains of Aspergillus unguis
Source: Pharmaceuticals (Basel). 2022 Jan 6;15(1):74. doi: 10.3390/ph15010074 (PMC8779881; doi:10.3390/ph15010074)
Supplement: Supplementary file 1 [file pharmaceuticals-15-00074-s001.zip › pharmaceuticals-1521034-supplementary.pdf]

Anti-Bacterial and Cytotoxic Phenolic Polyketides from Two Marine-Derived Fungal Strains of *Aspergillus unguis*

Cao Van Anh [1,2], Joo-Hee Kwon [3], Jong Soon Kang [3], Hwa-Sun Lee [1], Chang-Su Heo[1,2] and Hee Jae Shin [1,2,*]

[1] Marine Natural Products Chemistry Laboratory, Korea Institute of Ocean Science and Technology, 385 Haeyang-ro, Yeongdo-gu, Busan 49111, Korea; caovananh@kiost.ac.kr (C.V.A.); hwasunlee@kiost.ac.kr (H.-S.L.); science30@kiost.ac.kr (C.-S.H.)

[2] Department of Marine Biotechnology, University of Science and Technology (UST), 217 Gajungro, Yuseong-gu, Daejeon 34113, Korea

[3] Laboratory Animal Resource Center, Korea Research Institute of Bioscience and Biotechnology, 30 Yeongudanjiro, Cheongju 28116, Korea; juhee@kribb.re.kr (J.-H.K.); kanjon@kribb.re.kr (J.S.K)

* Correspondence: shinhj@kiost.ac.kr; Tel.: +82-51-664-3341; Fax: +82-51-664-3340

Contents

Figure S1. Structures of **1-17** isolated from *A. unguis* IV17-109 and 158SC-067 (detailed). 3
Figure S2. Comparison of chemical shifts of the new compounds (**1**, **3**, and **12**) with those of their analogs. 4
Figure S3. HRESIMS data of **1**. 5
Figure S4. ^{1}H NMR spectrum of **1**. 6
Figure S5. ^{13}C NMR spectrum of **1**. 6
Figure S6. HSQC spectrum of **1**. 7
Figure S7. ^{1}H-^{1}H COSY spectrum of **1**. 8
Figure S8. HMBC spectrum of **1**. 9
Figure S9. NOESY spectrum of **1**. 10
Figure S10. HRESIMS data of **3**. 11
Figure S11. ^{1}H NMR spectrum of **3**. 12
Figure S12. ^{13}C NMR spectrum of **3**. 12
Figure S13. HSQC spectrum of **3**. 13
Figure S14. ^{1}H-^{1}H COSY spectrum of **3**. 14
Figure S15. HMBC spectrum of **3**. 15
Figure S16. NOESY spectrum of **3**. 16
Figure S17. HRESMS data of **12**. 17
Figure S18. ^{1}H NMR spectrum of **12**. 18
Figure S19. ^{13}C NMR spectrum of **12**. 18
Figure S20. HSQC spectrum of **12**. 19
Figure S21. ^{1}H-^{1}H COSY spectrum of **12**. 20
Figure S23. NOESY spectrum of **12**. 22
Figure S24. Comparison of optical rotation signs between **12** and other glycerides of carboxylic acids.. 24
Figure S25. ^{1}H NMR spectrum of decarboxyunguidepside A (**2**). 25
Figure S26. ^{13}C NMR spectrum of decarboxyunguidepside A (**2**). 25
Figure S27. ^{1}H NMR spectrum of **4**. 26
Figure S29. ^{1}H NMR spectrum of **11**. 27
Figure S30. ^{13}C NMR spectrum of **11**. 27
Figure S31. Results of the cytotoxicity test of compounds **1-11** and **13-16**. 30

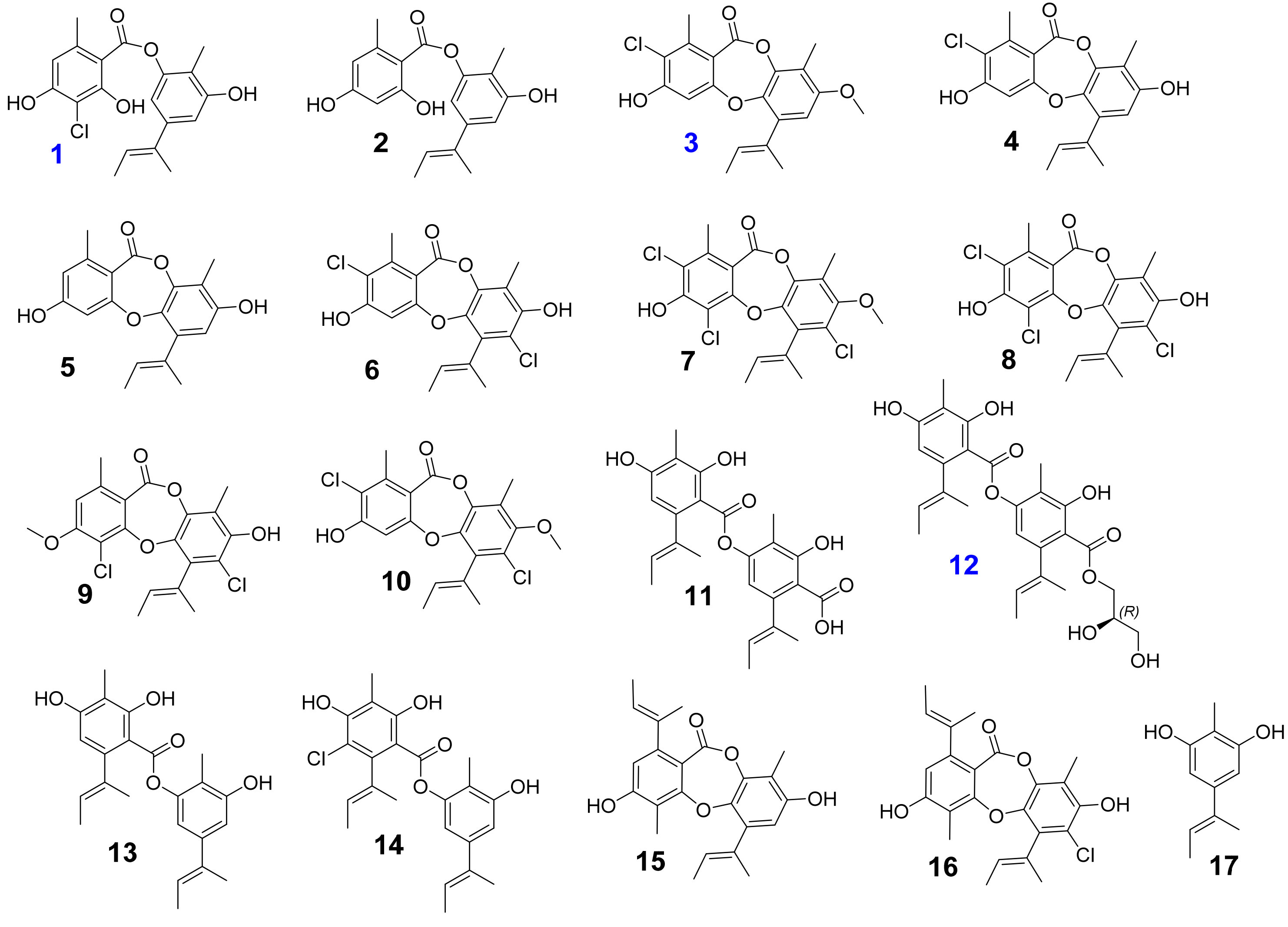

Figure S1. Structures of **1-17** isolated from *A. unguis* IV17-109 and 158SC-067 (detailed).

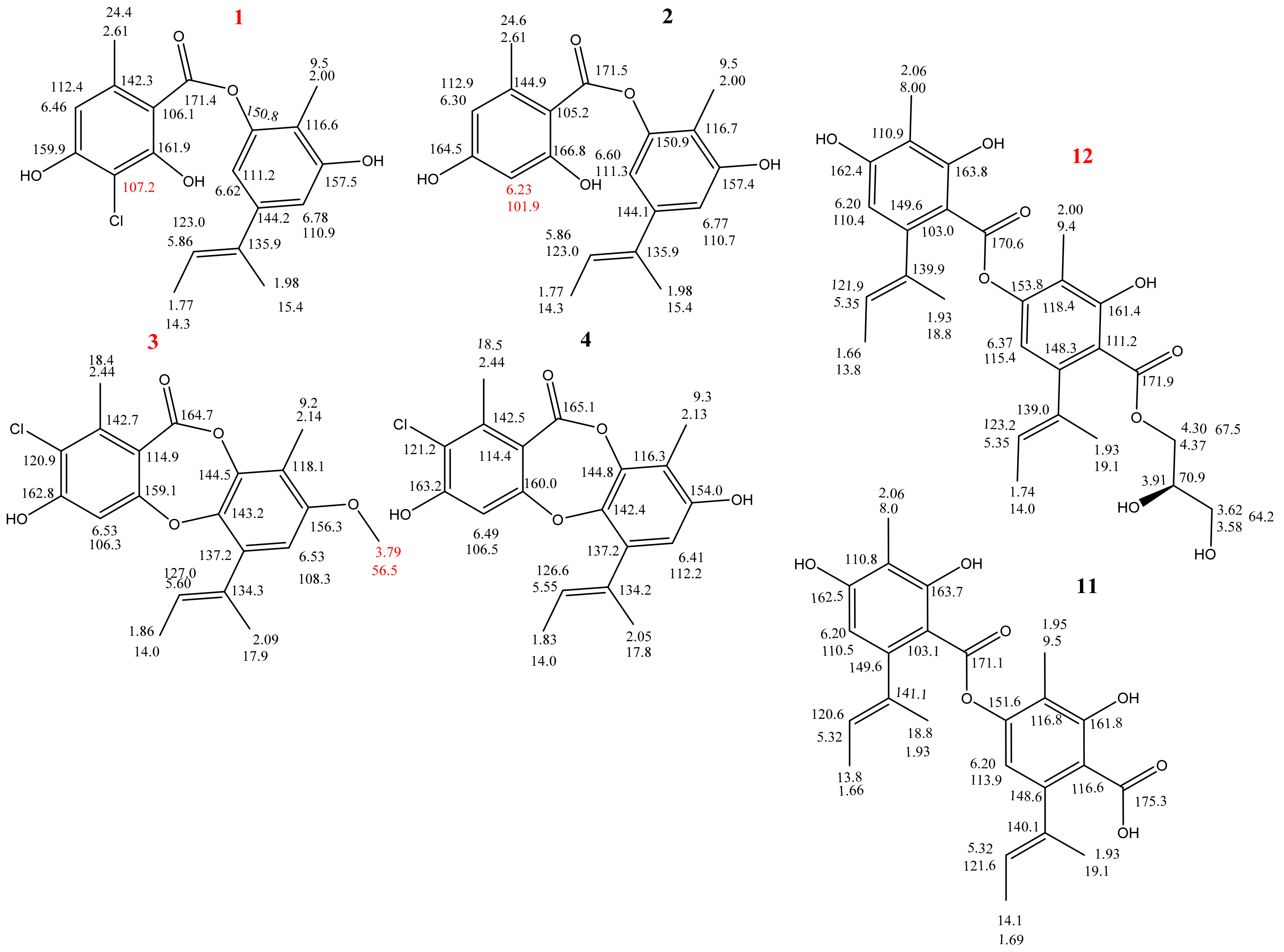

Figure S2. Comparison of chemical shifts of the new compounds (**1**, **3**, and **12**) with those of their analogs.

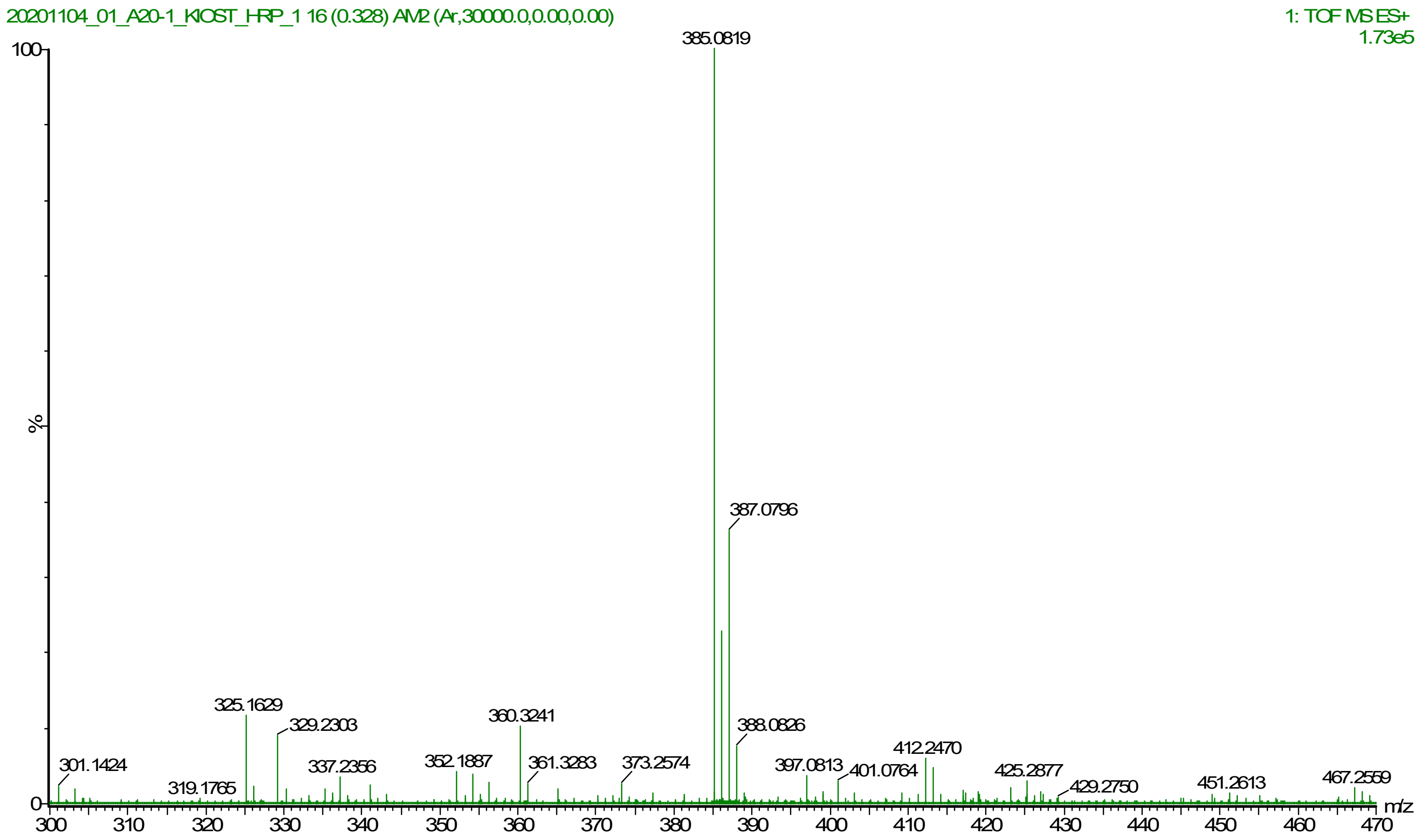

Minimum:			-1.5					
Maximum:		100.0 5.0	100.0					
Mass	Calc. Mass	mDa	PPM	DBE	i-FIT	Norm	Conf(%)	Formula
385.0819	385.0819	0.0	0.0	9.5	909.7	n/a	n/a	C19 H19 O5 Na Cl

Figure S3. HRESIMS data of **1**.

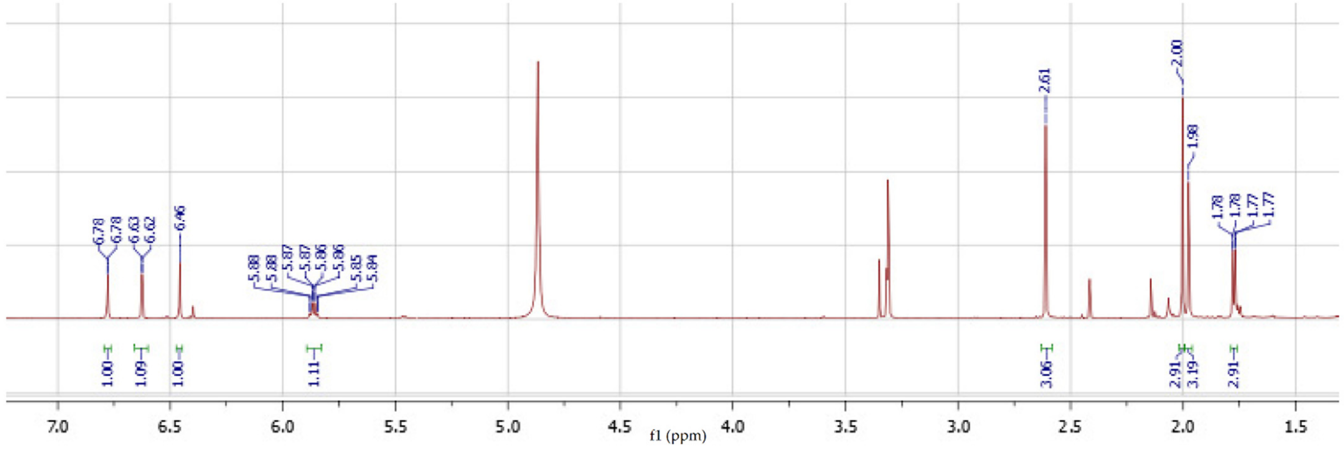

Figure S4. ^{1}H NMR spectrum of **1**.

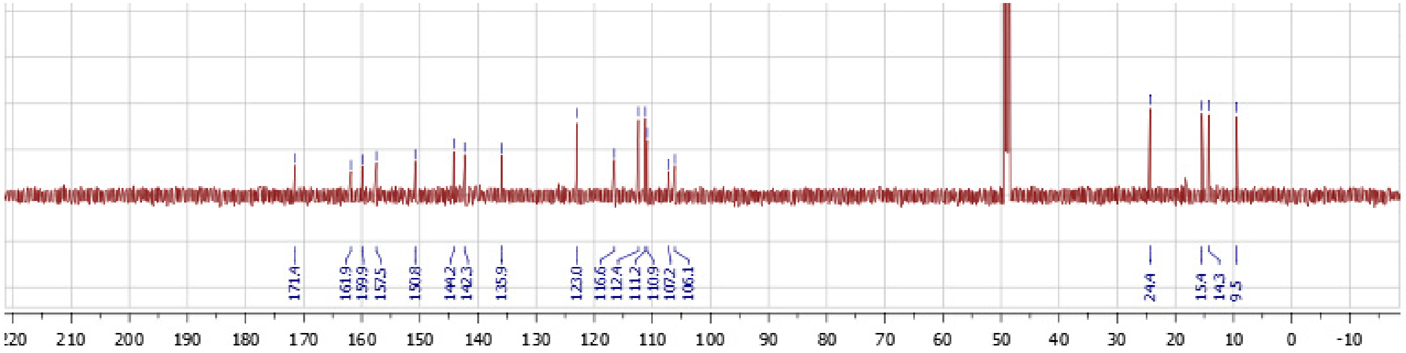

Figure S5. ^{13}C NMR spectrum of **1**.

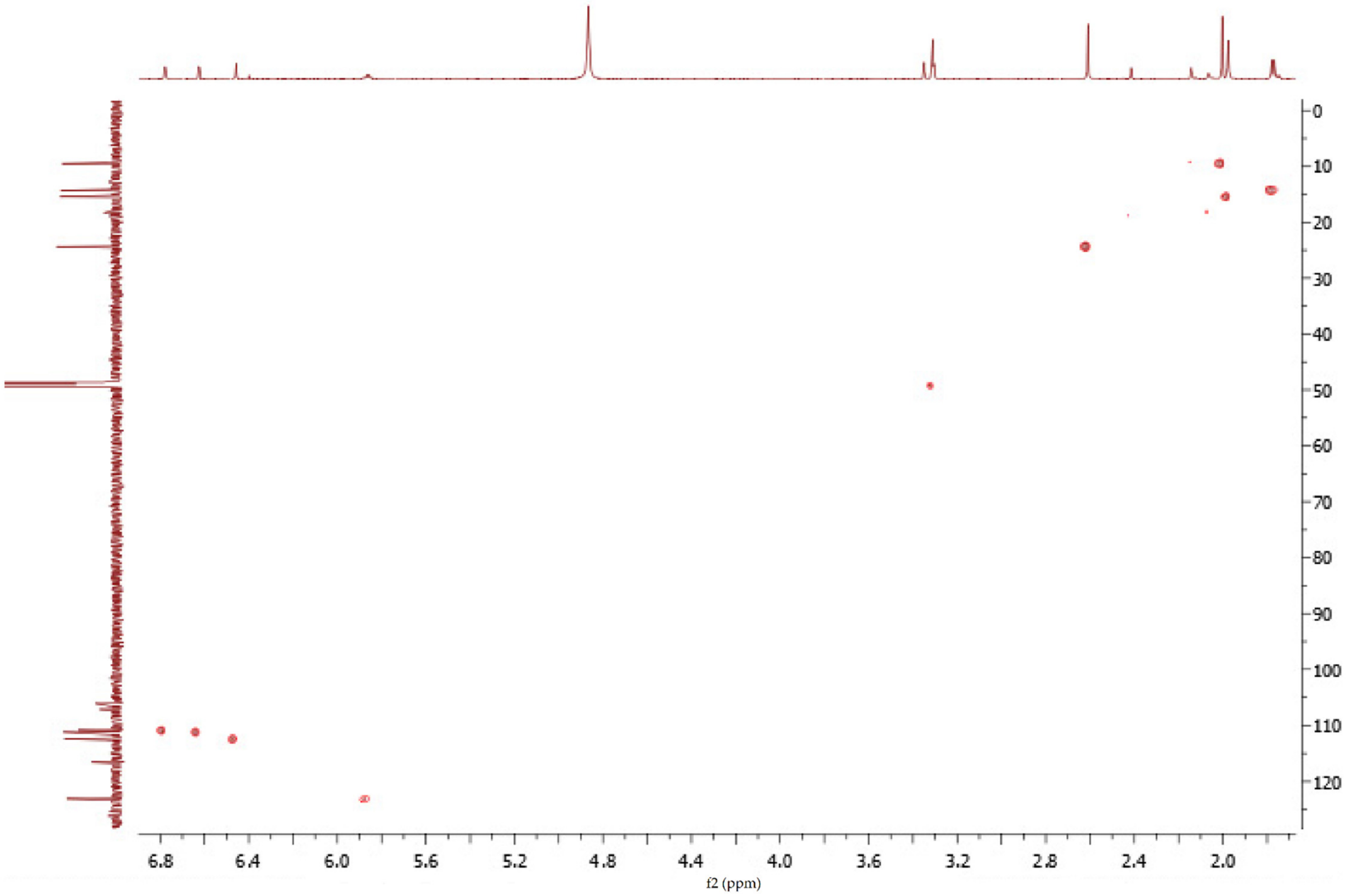

Figure S6. HSQC spectrum of **1**.

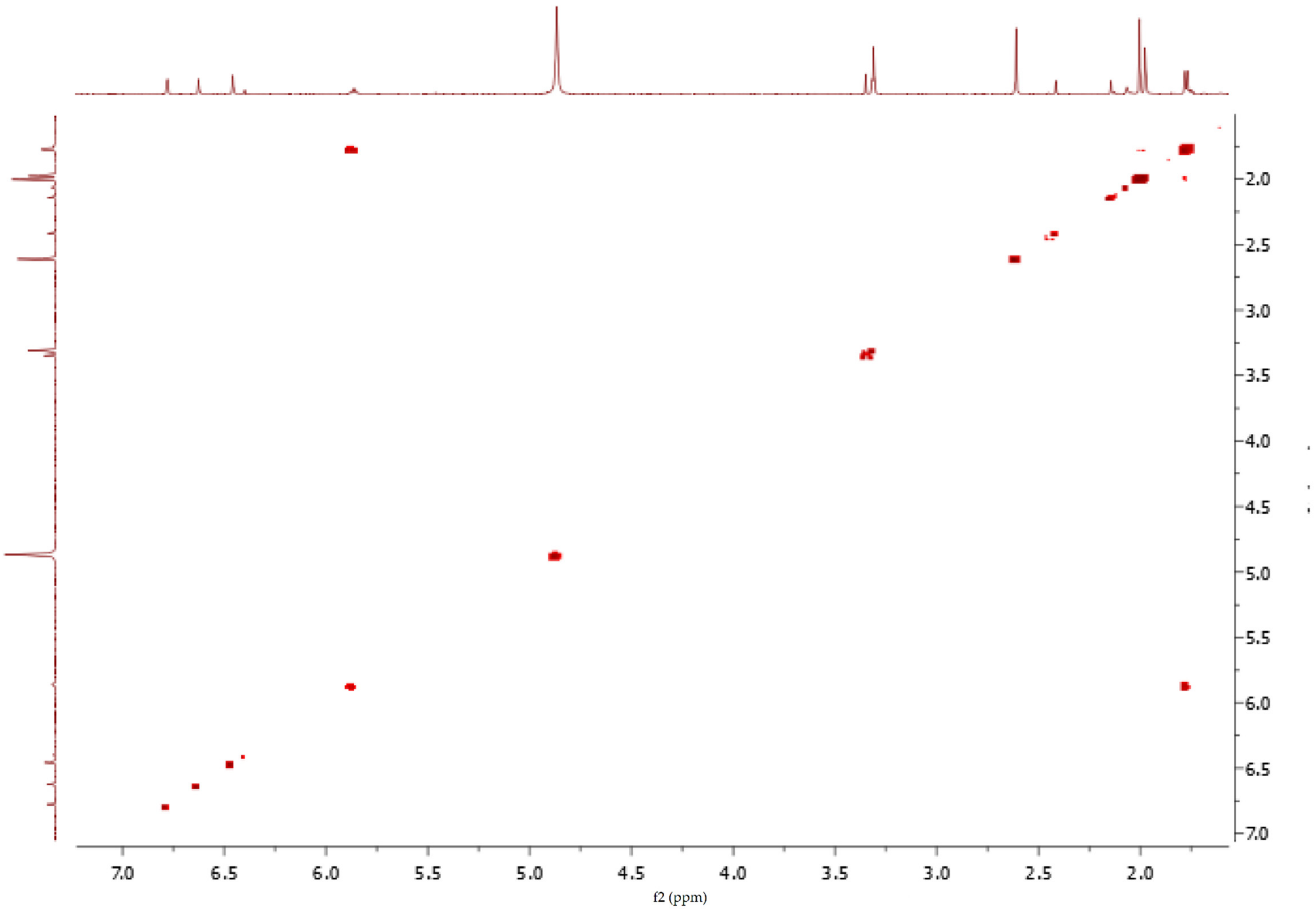

Figure S7. ^{1}H-^{1}H COSY spectrum of **1**.

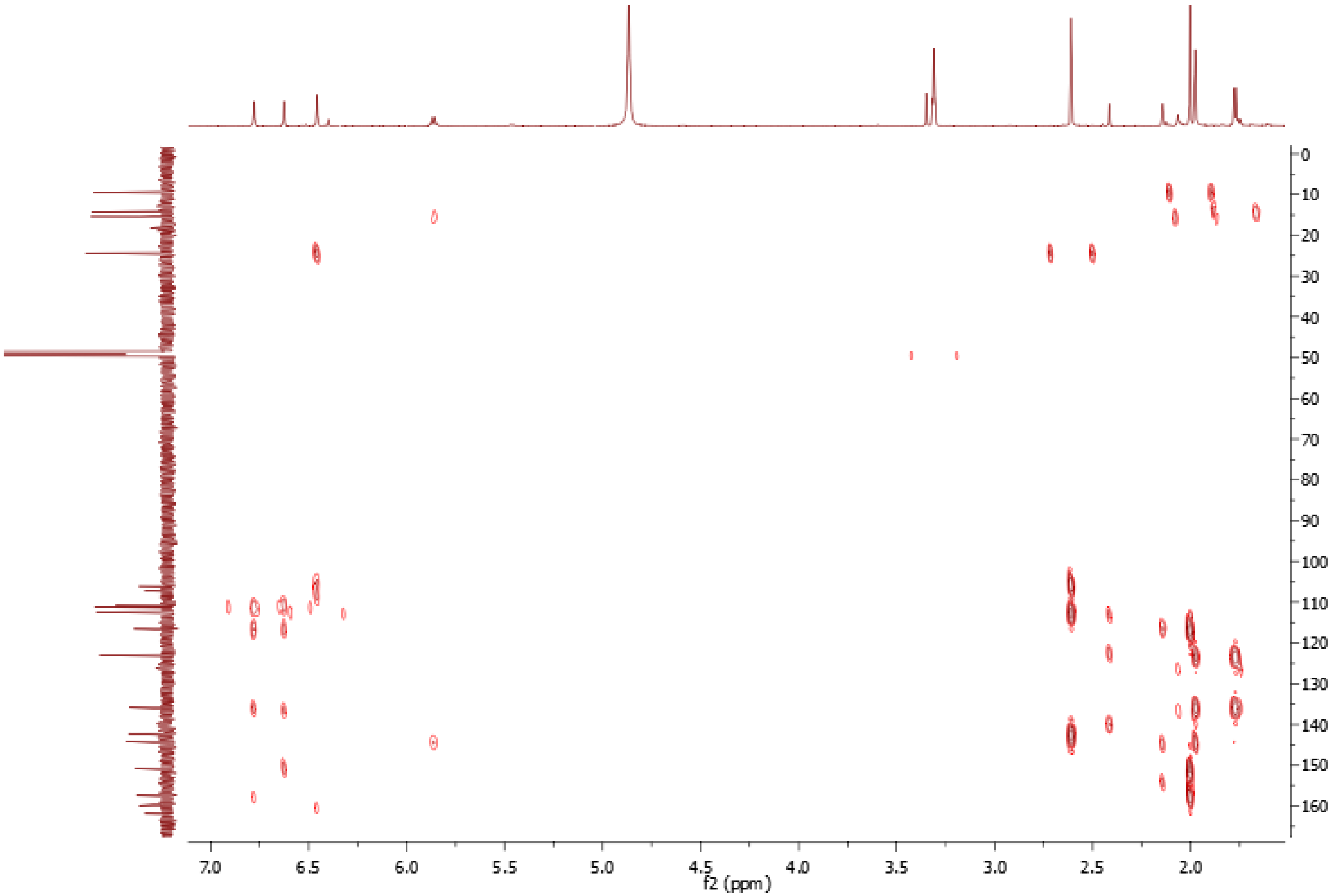

Figure S8. HMBC spectrum of **1**.

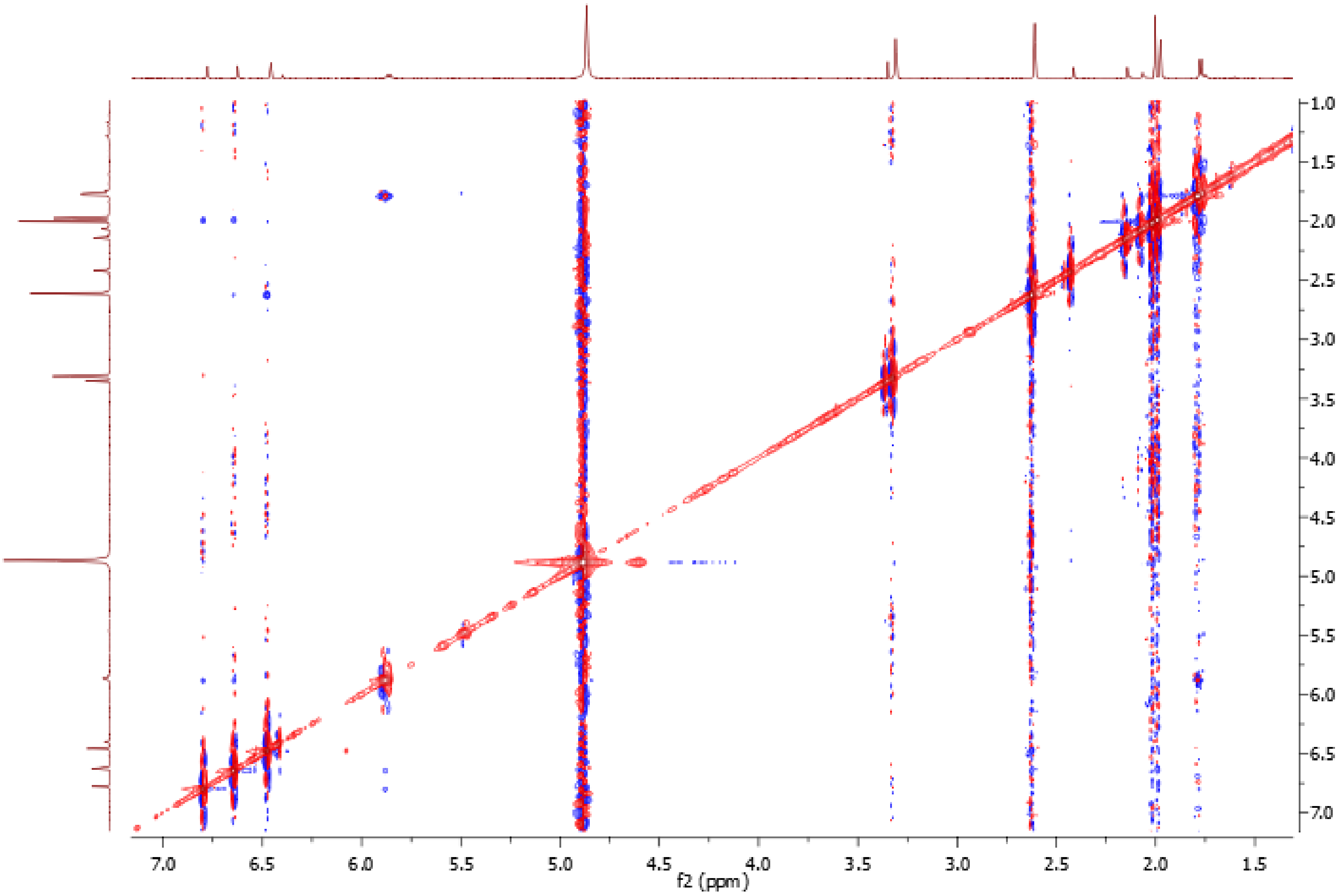

Figure S9. NOESY spectrum of **1**.

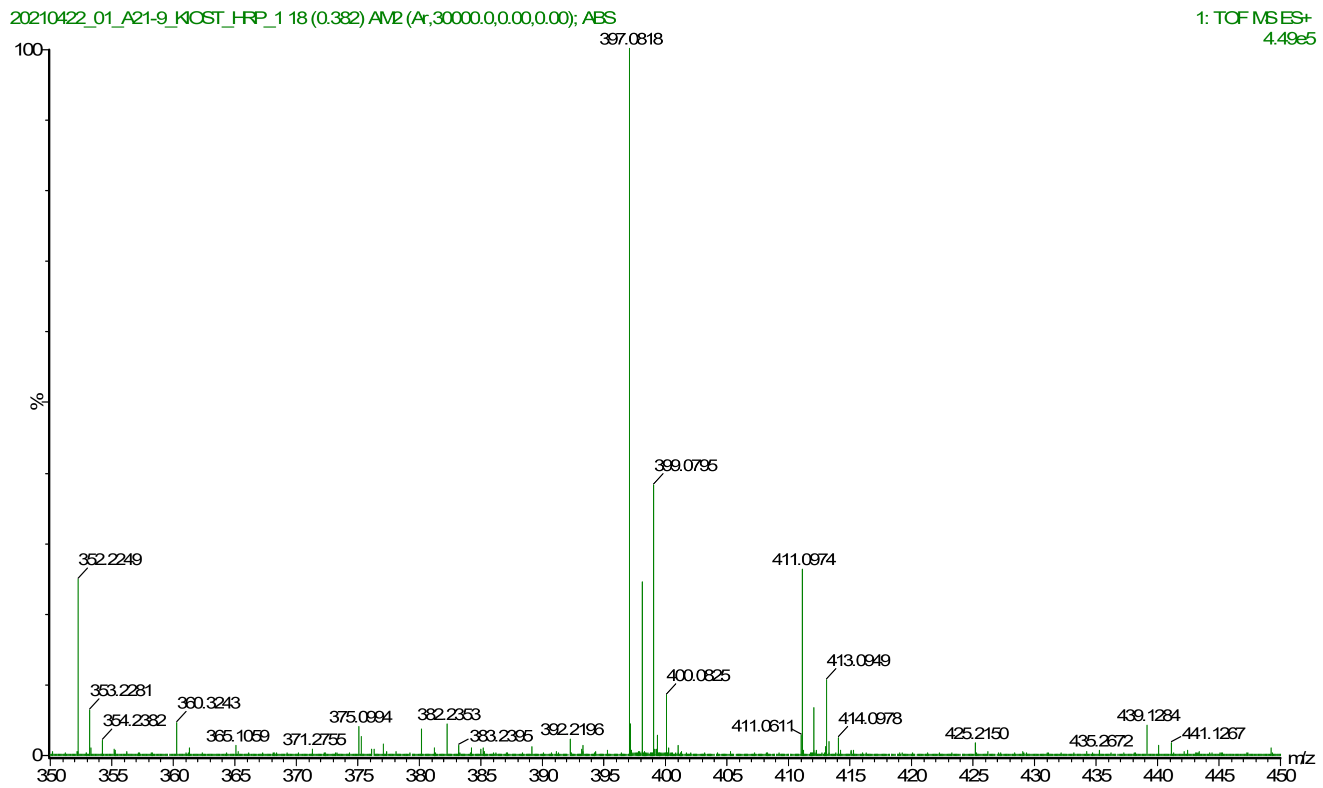

C: 0-30 H: 0-50 O: 0-5 Na: 0-1 Cl: 0-1

	Mass	Calc. Mass	mDa	PPM	DBE	i-FIT	Norm	Conf(%)	Formula
Minimum:				-1.5					
Maximum:		100.0	5.0	100.0					
	397.0818	397.0819	-0.1	-0.3	10.5	919.6	n/a	n/a	C20 H19 O5 Na Cl

Figure S10. HRESIMS data of **3**.

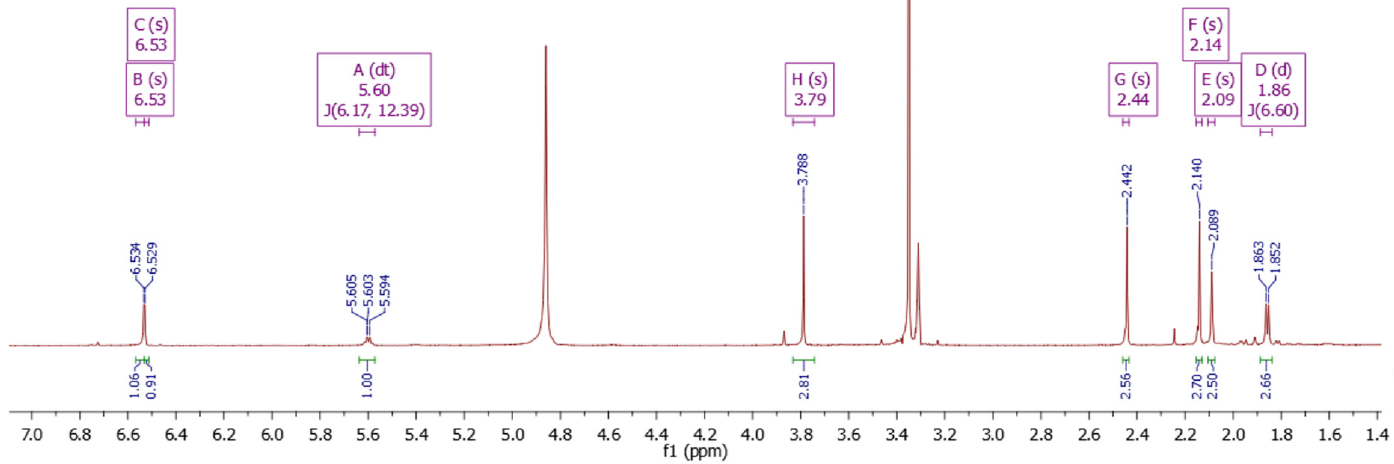

Figure S11. ^{1}H NMR spectrum of **3**.

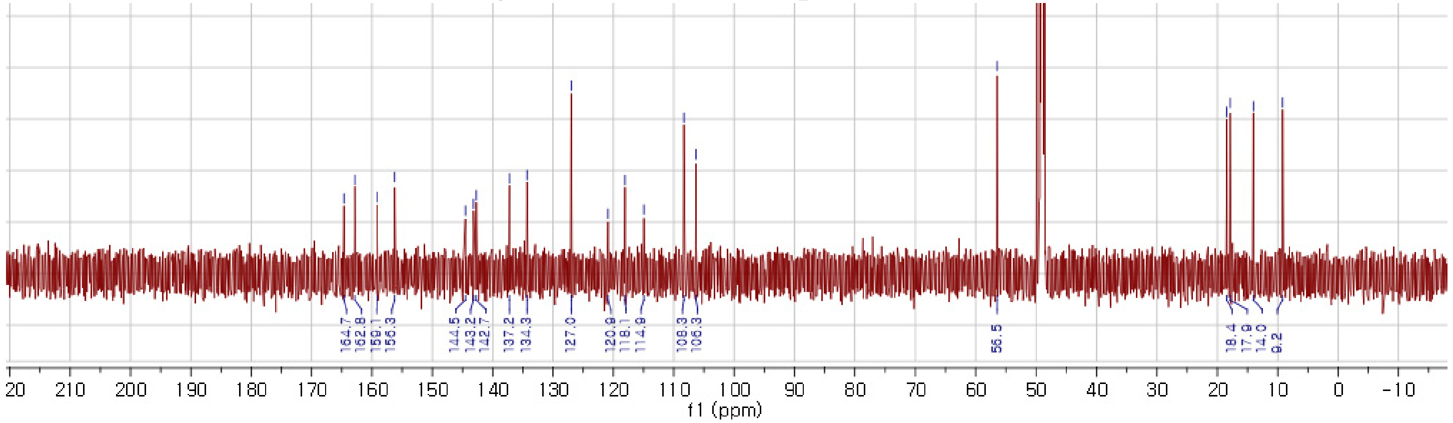

Figure S12. ^{13}C NMR spectrum of **3**.

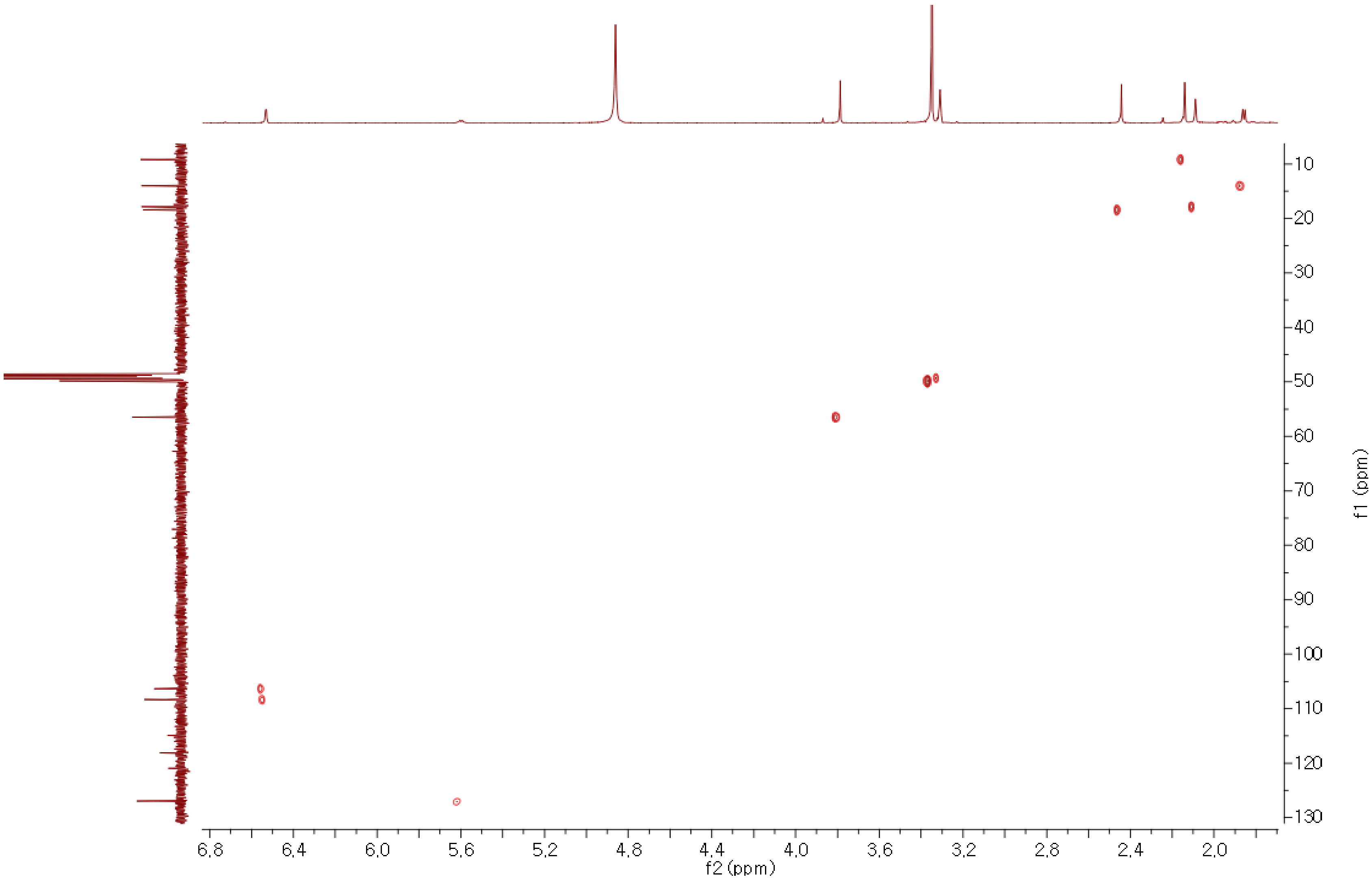

Figure S13. HSQC spectrum of **3**.

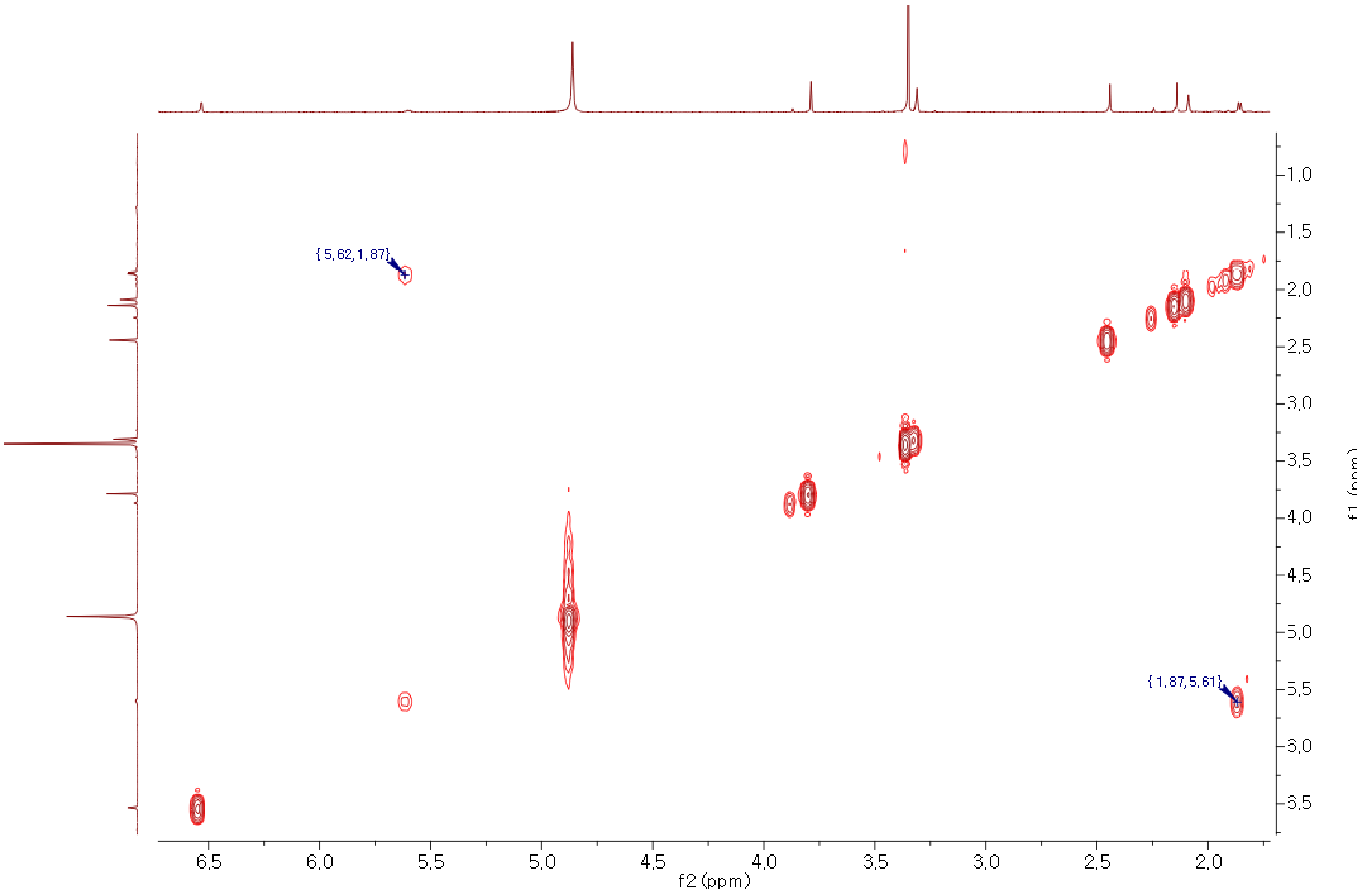

Figure S14. ^{1}H-^{1}H COSY spectrum of **3**.

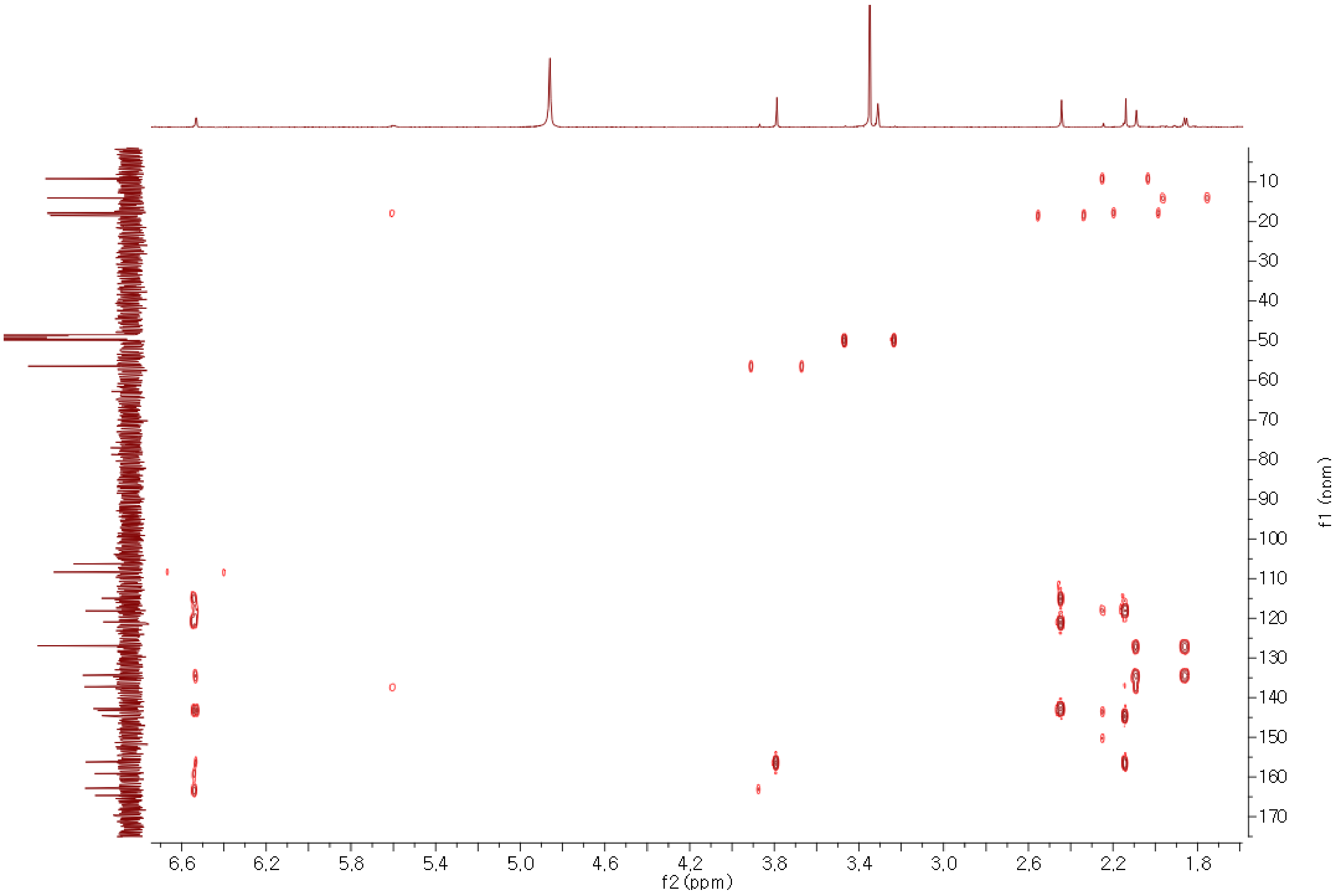

Figure S15. HMBC spectrum of **3**.

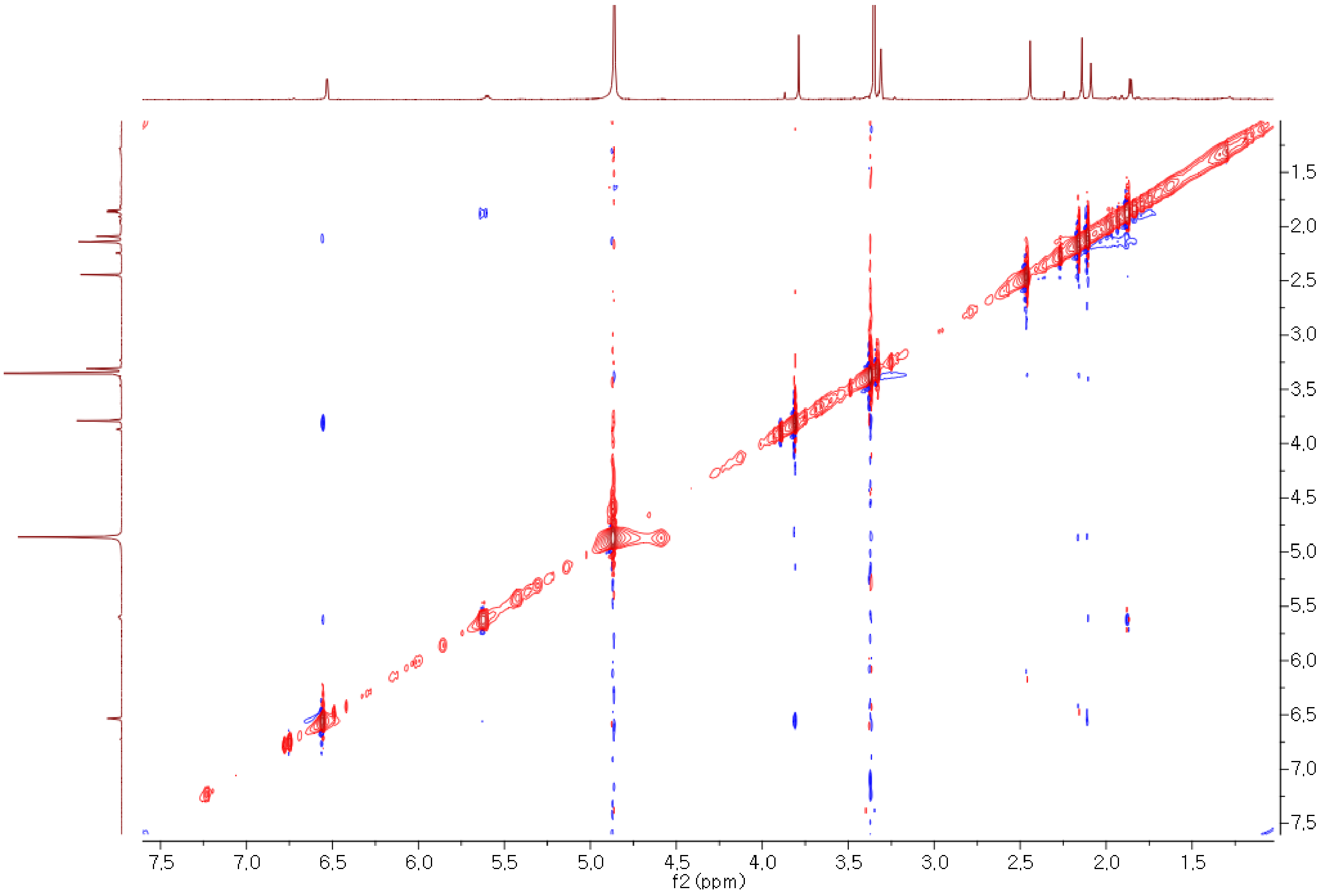

Figure S16. NOESY spectrum of **3**.

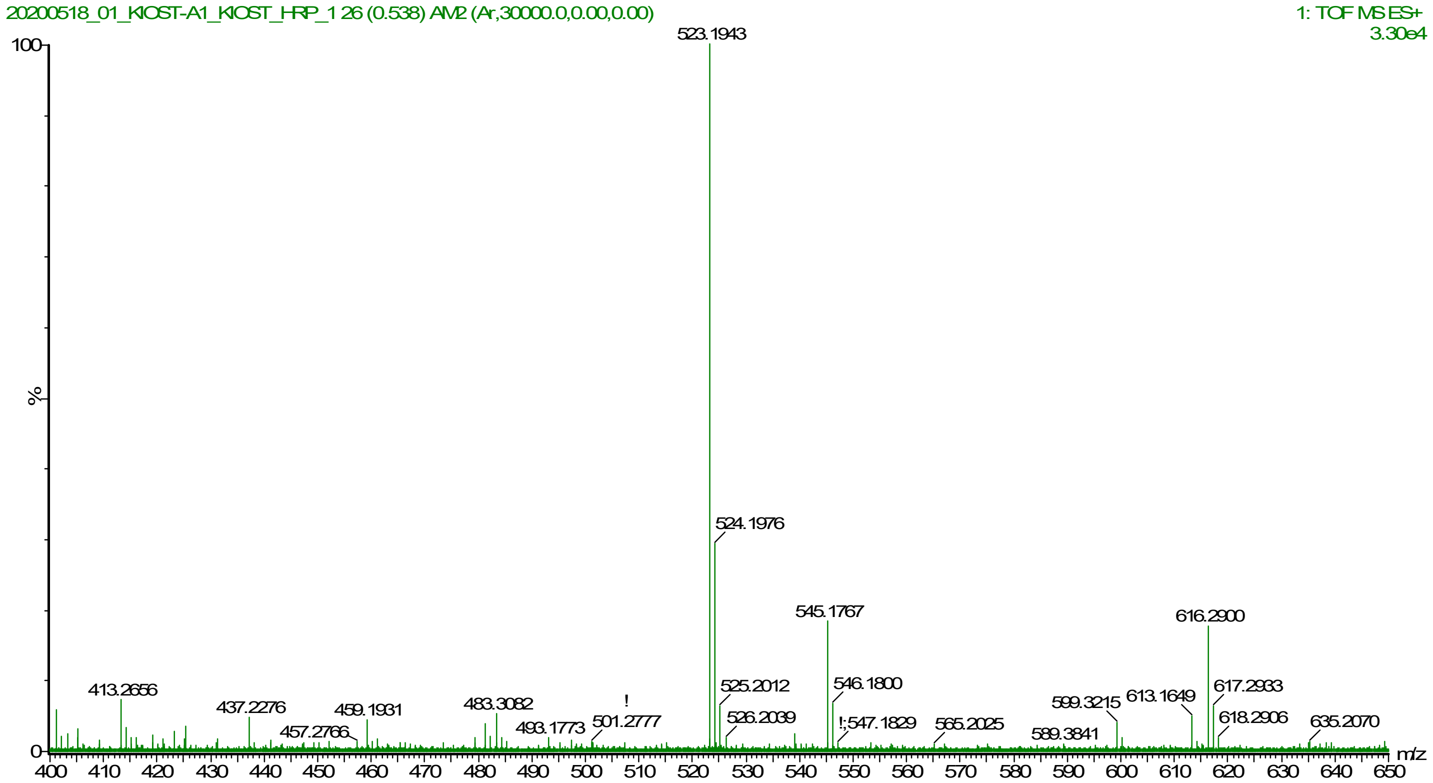

Elements Used:

C: 0-30 H: 0-35 O: 0-9 Na: 0-1 P: 0-1

Mass	Calc. Mass	mDa	PPM	DBE	i-FIT	Norm	Conf(%)	Formula
Minimum:				-1.5				
Maximum:		100.0	5.0	100.0				
523.1943	523.1944	-0.1	-0.2	11.5	562.4	0.001	99.86	C27 H32 O9 Na
	523.1968	-2.5	-4.8	14.5	569.0	6.570	0.14	C29 H31 O9

Figure S17. HRESMS data of **12**.

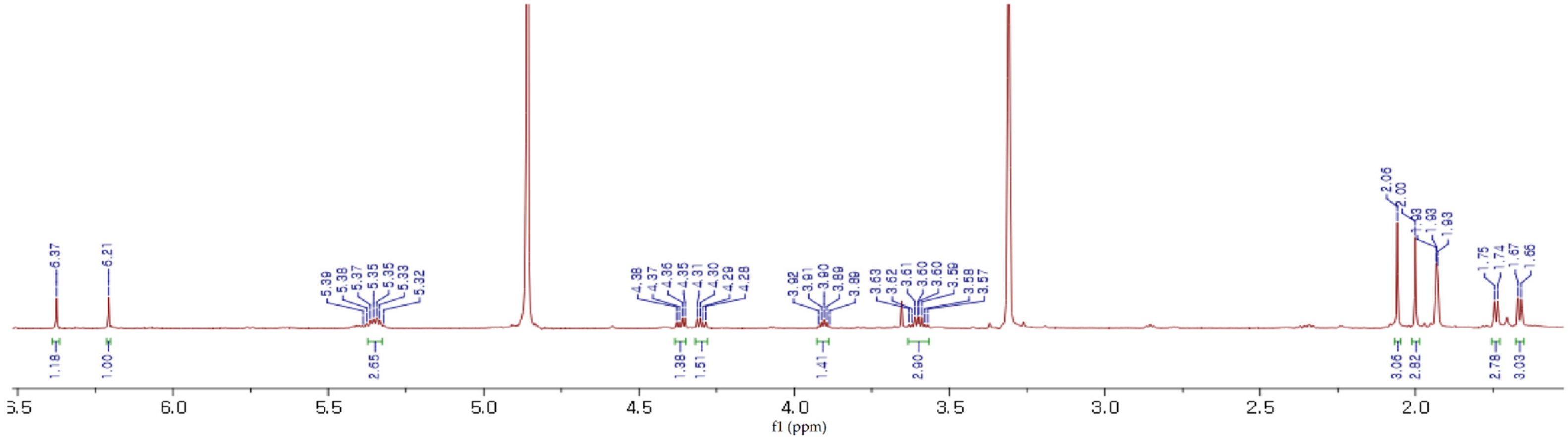

Figure S18. ^{1}H NMR spectrum of **12**.

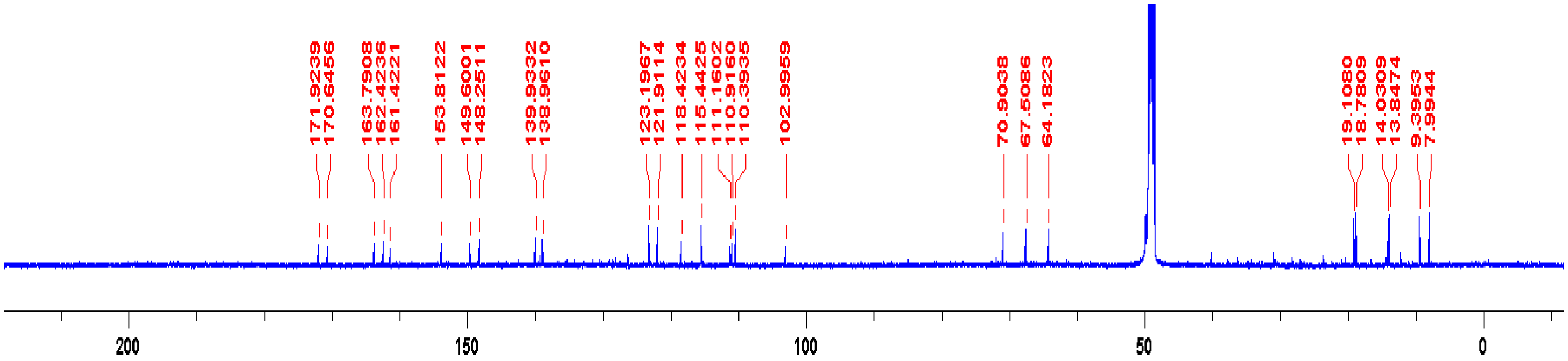

Figure S19. ^{13}C NMR spectrum of **12**.

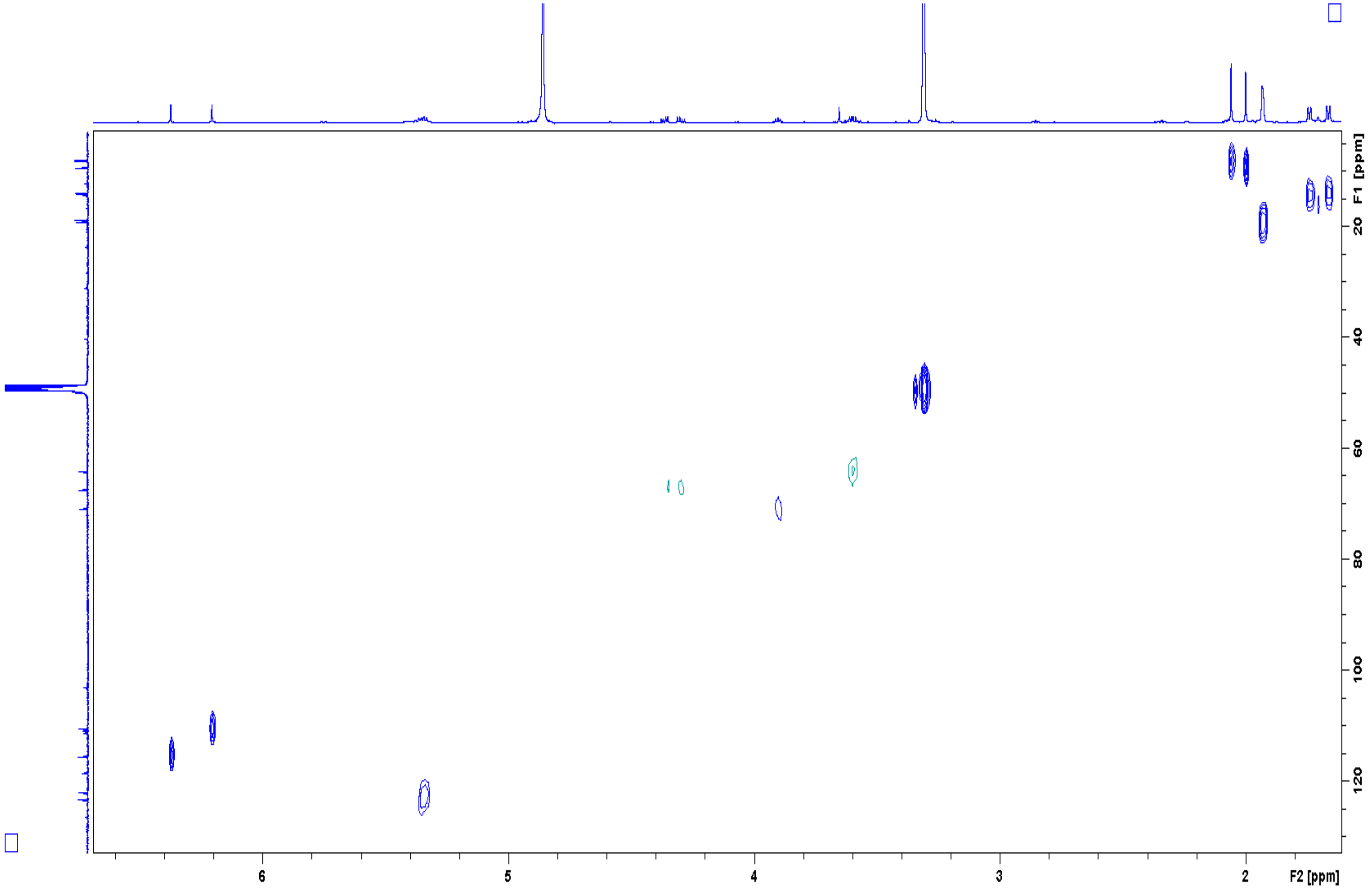

Figure S20. HSQC spectrum of **12**.

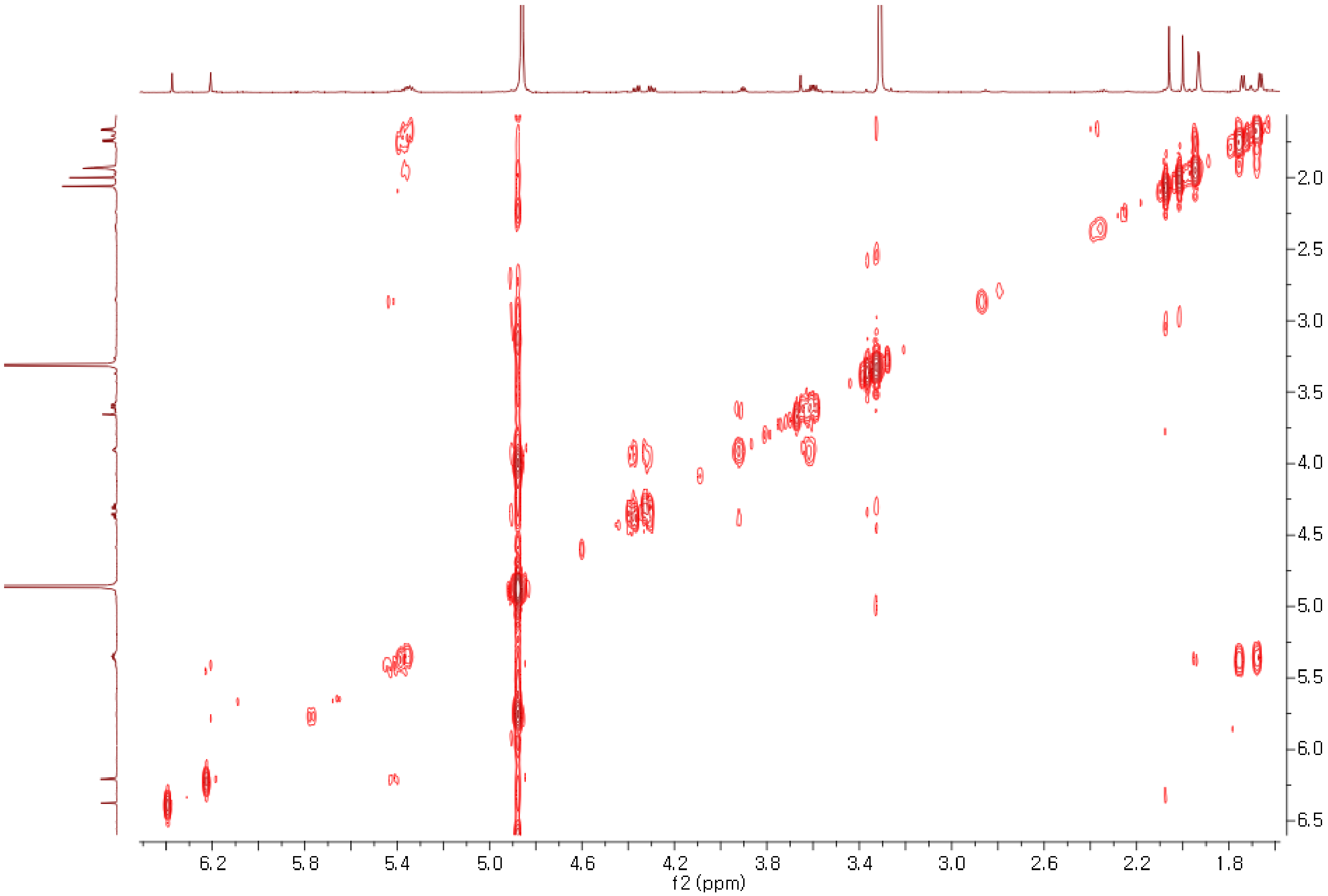

Figure S21. ^{1}H-^{1}H COSY spectrum of **12**.

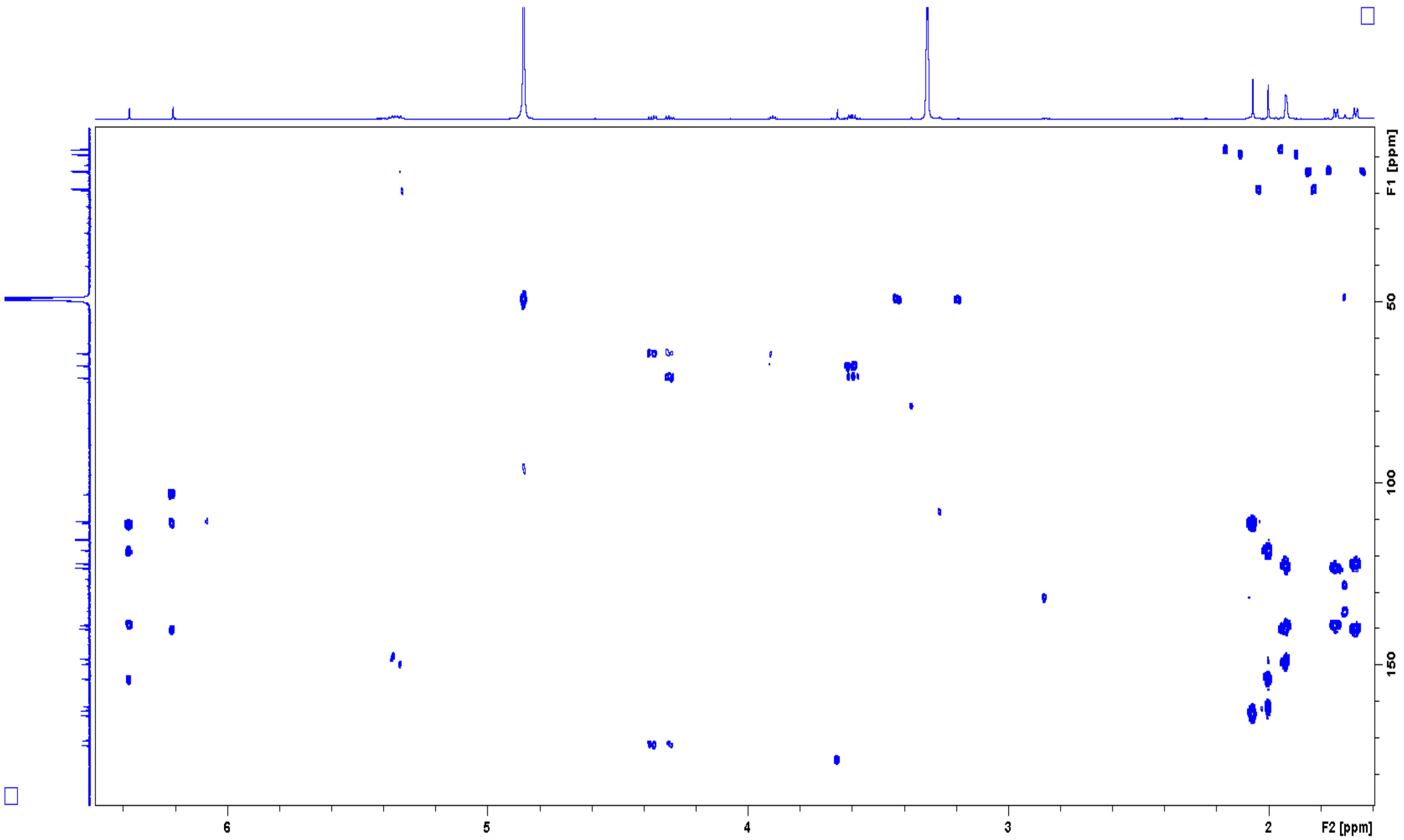

Figure S22. HMBC spectrum of **12.**

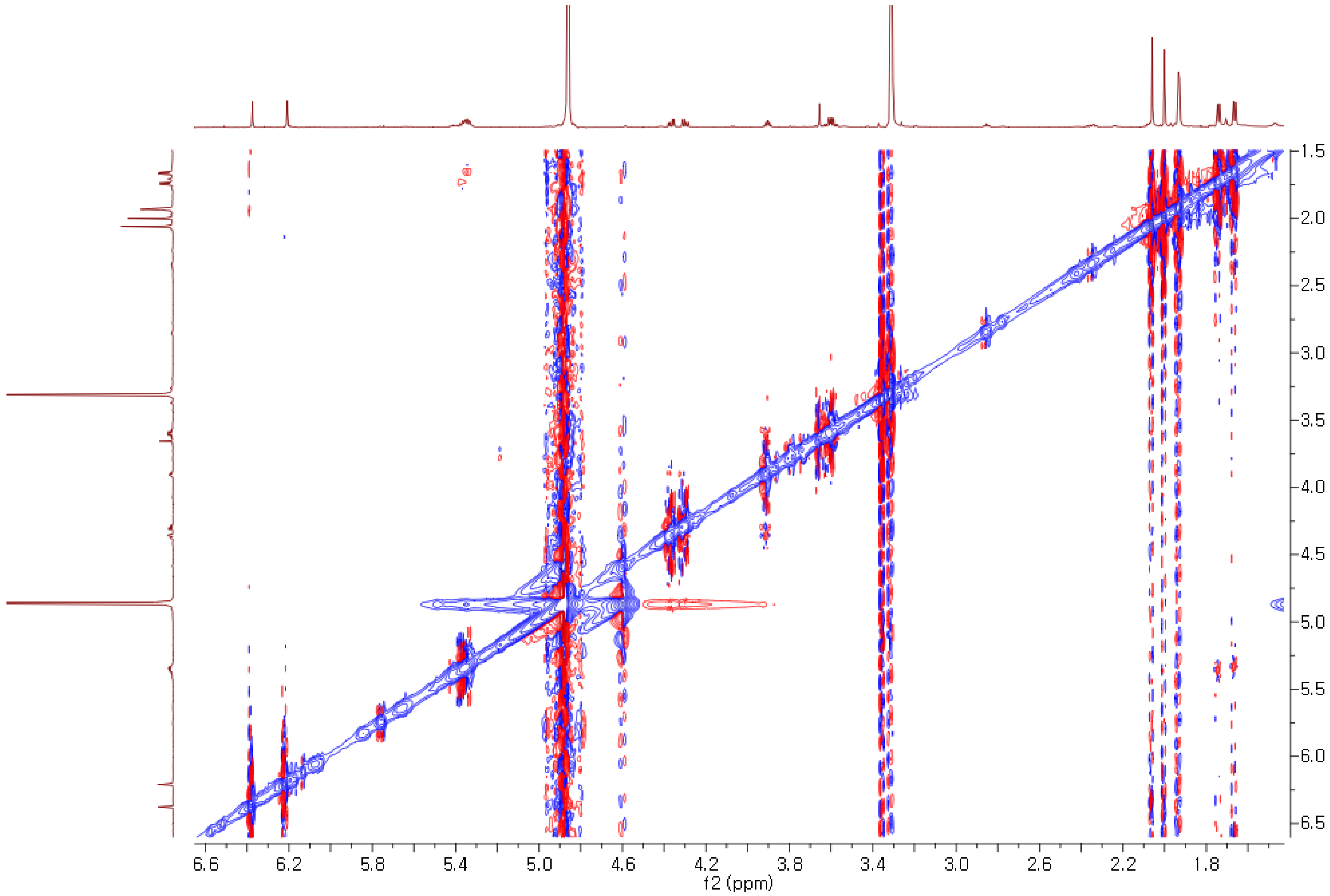

Figure S23. NOESY spectrum of **12**.

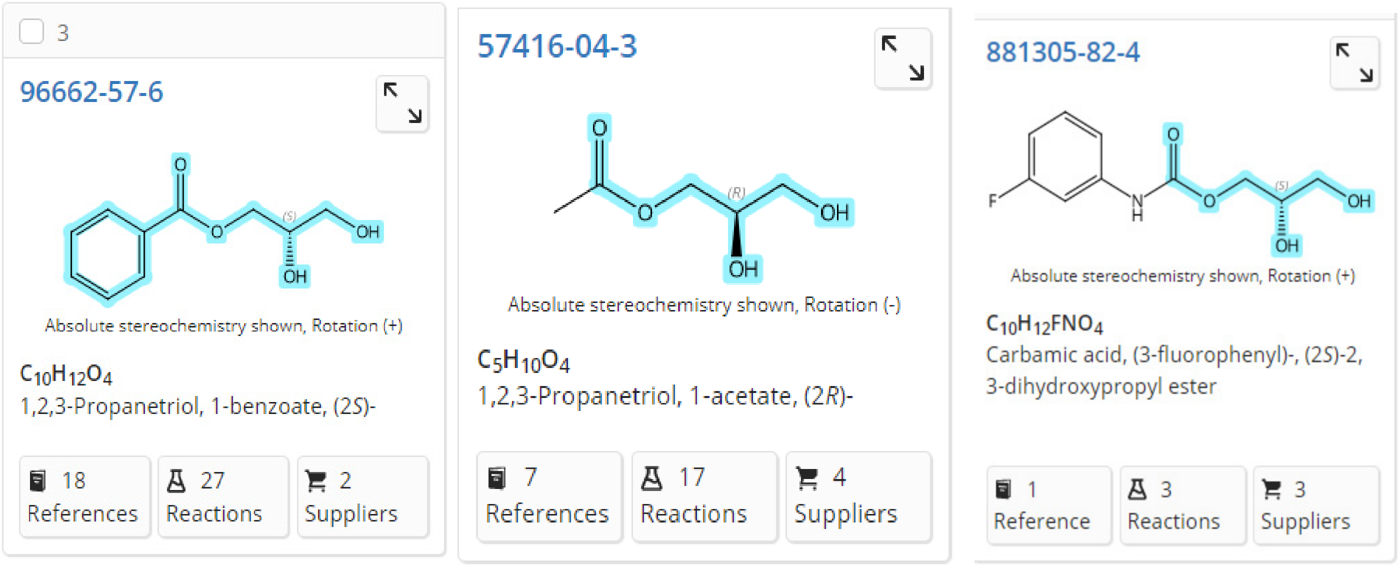

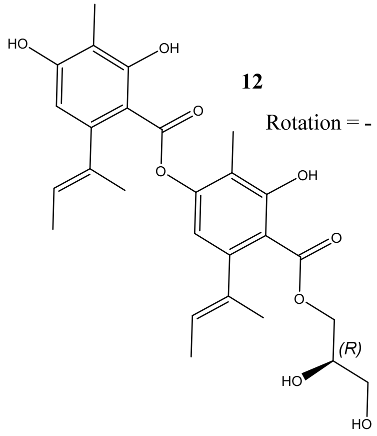

12

Rotation = - 10

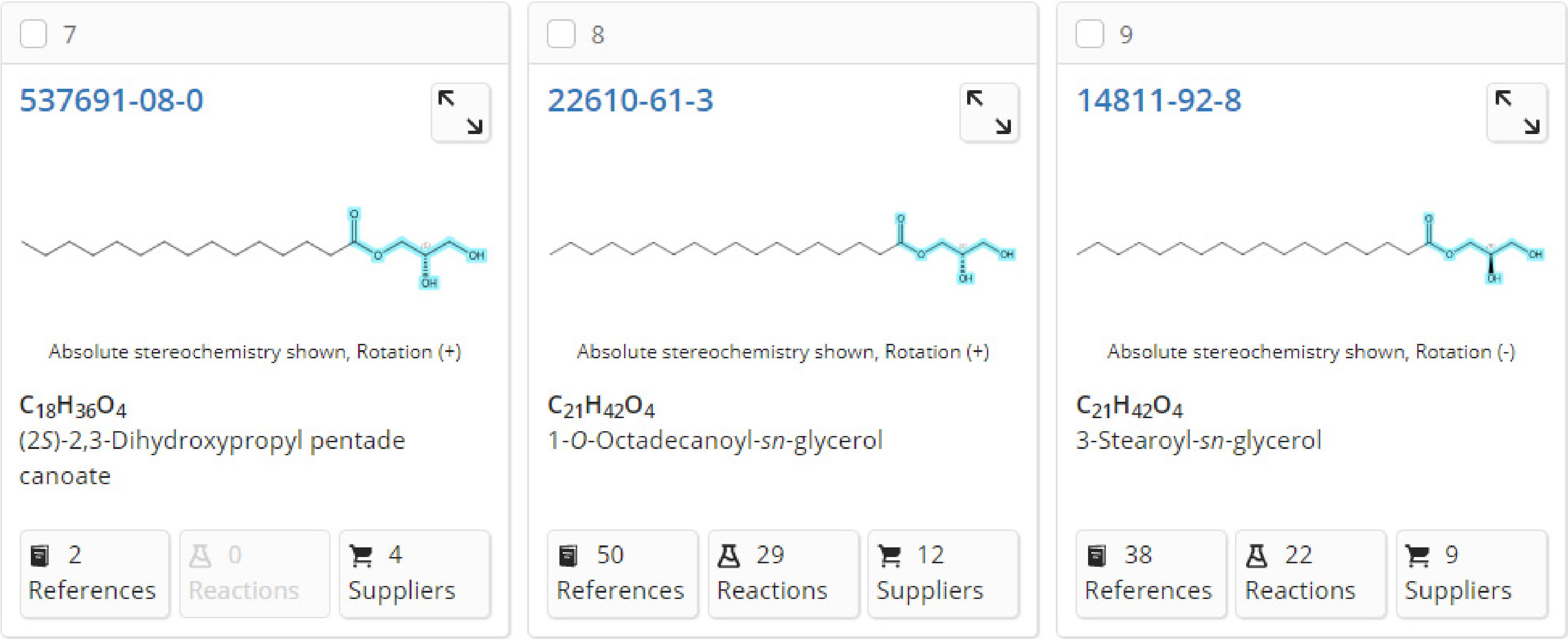

Figure S24. Comparison of optical rotation signs between **12** and other glycerides of carboxylic acids.

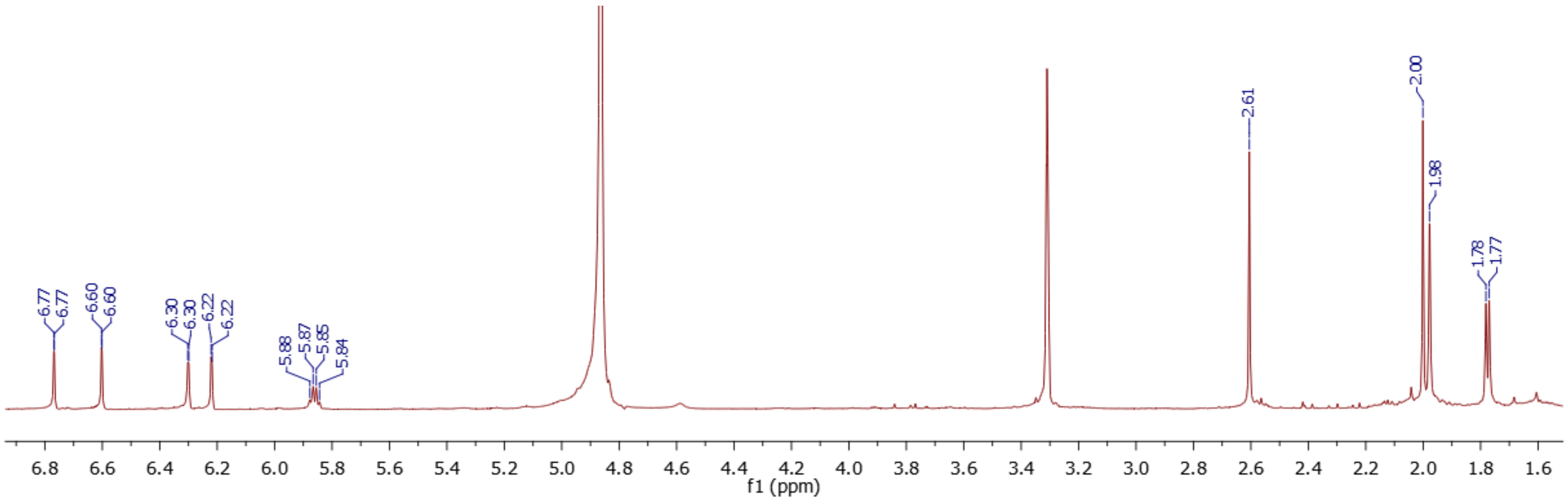

Figure S25. ^{1}H NMR spectrum of decarboxyunguidepside A (**2**).

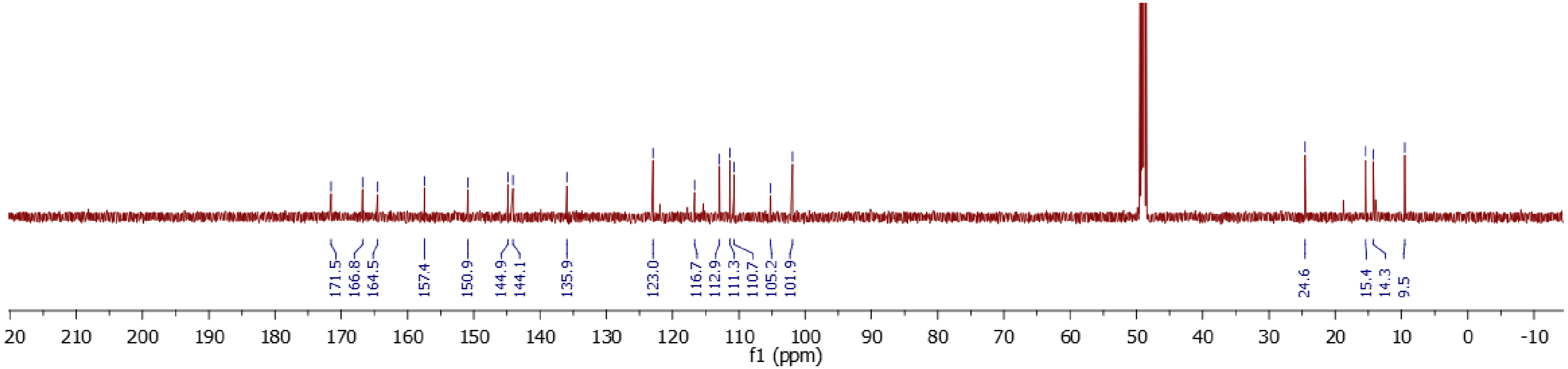

Figure S26. ^{13}C NMR spectrum of decarboxyunguidepside A (**2**).

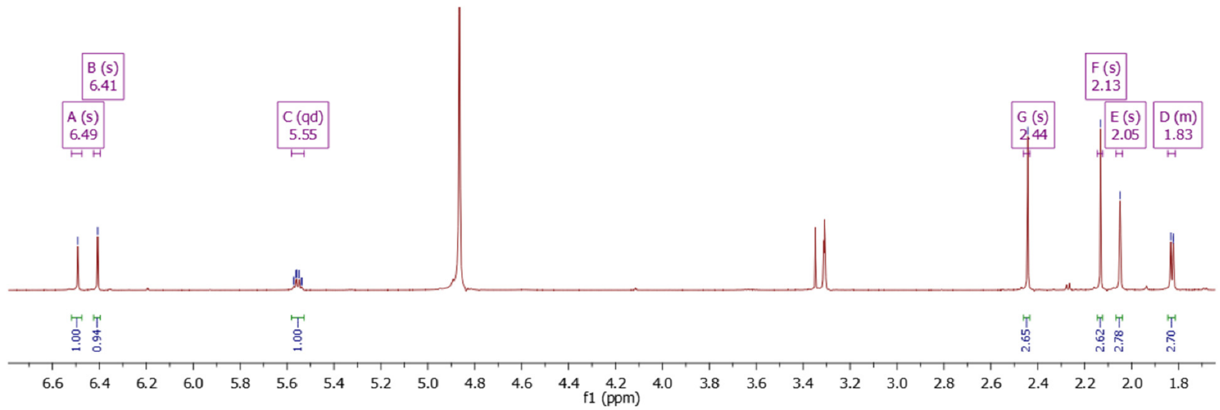

Figure S27. ^{1}H NMR spectrum of **4**.

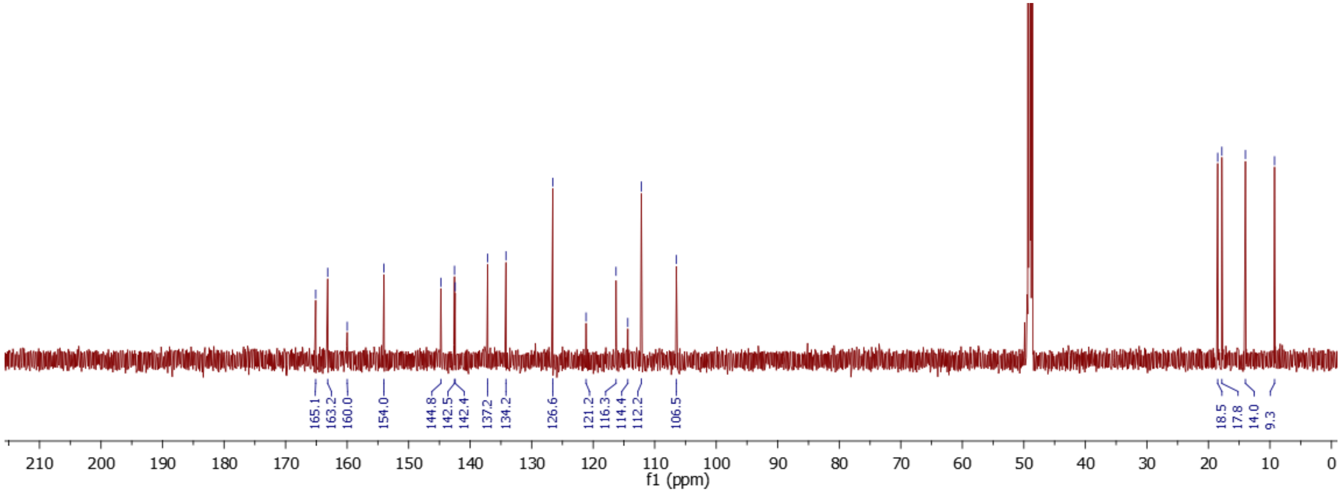

Figure S28. ^{13}C NMR spectrum of **4**.

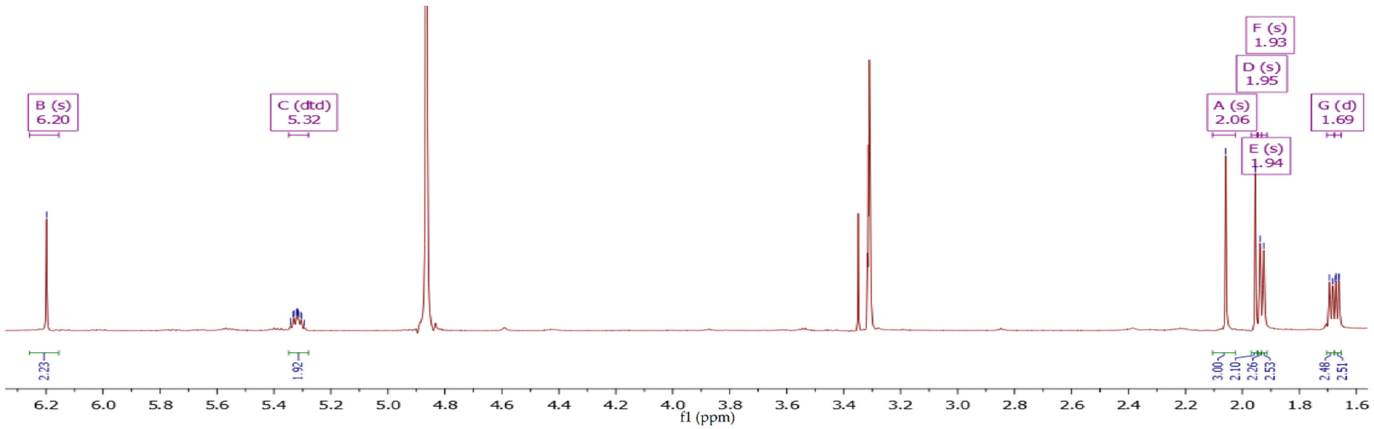

Figure S29. ^{1}H NMR spectrum of **11**.

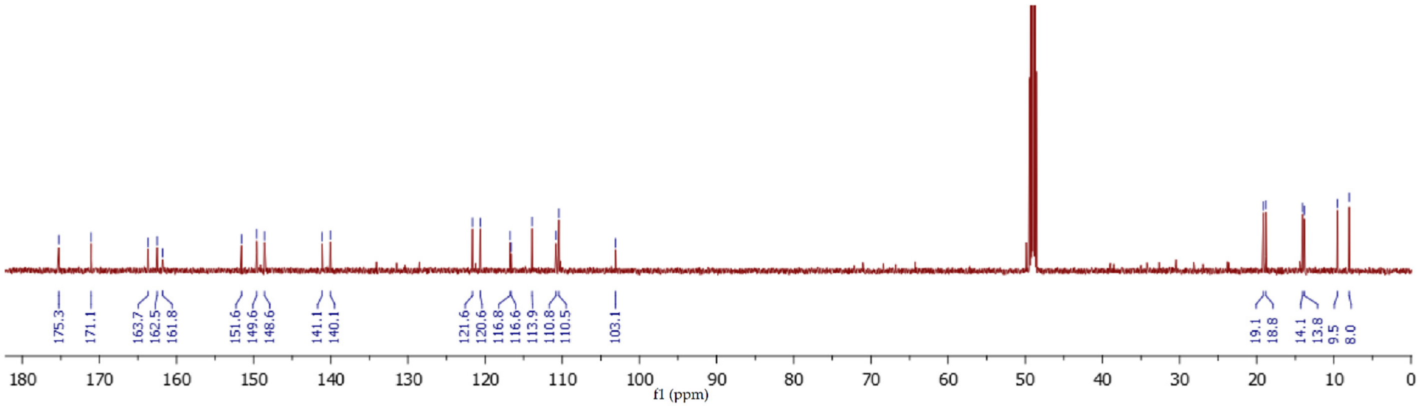

Figure S30. ^{13}C NMR spectrum of **11**.

ACHN

Compound	1		2		3		4		5		6		7		8	
Conc.(ug/ml)	Mean	SD	Mean	SD	Mean	SD	Mean	SD	Mean	SD	Mean	SD	Mean	SD	Mean	SD
30	-11.74	6.98	-27.25	6.19	-20.33	2.54	-28.45	4.20	-25.45	7.14	-20.04	9.88	0.22	2.87	-22.62	5.41
10	21.50	8.31	-15.83	8.58	19.32	4.76	-5.75	3.46	-12.08	2.34	-8.63	6.06	70.92	7.86	23.46	3.72
3	75.37	3.45	23.72	5.09	75.00	9.16	13.33	6.59	27.91	3.44	22.02	7.15	84.61	3.44	60.17	6.11
1	91.86	5.91	37.88	9.23	92.13	4.04	82.94	4.58	70.20	6.80	86.25	5.31	91.20	4.08	72.23	2.06
0.3	99.47	1.89	85.18	5.50	100.24	4.56	91.39	5.73	94.53	5.81	95.58	6.07	100.61	4.78	90.34	1.19
GI50 (ug/ml)	5.043		0.820		4.842		1.640		1.620		1.907		12.27		3.150	
GI50 (uM)	13.9		2.5		12.9		4.6		5.0		4.8		27.7		7.3	

Compound	9		10		11		13		14		15		16		Adriamycin	
Conc.(ug/ml)	Mean	SD	Mean	SD	Mean	SD	Mean	SD	Mean	SD	Mean	SD	Mean	SD	Mean	SD
30	84.87	4.21	15.20	6.82	33.00	7.62	-18.46	5.90	-10.47	2.58	-4.03	8.68	-6.93	4.80	-14.99	2.94
10	87.52	4.85	76.50	5.46	73.04	3.28	-10.47	6.90	24.35	9.81	24.06	7.94	31.15	4.64	-8.79	3.78
3	91.65	1.78	90.23	1.66	87.60	2.38	11.60	6.28	79.55	8.18	79.86	4.15	86.40	5.11	10.69	5.73
1	97.32	4.75	93.08	4.27	96.92	1.04	52.30	4.28	90.51	2.88	91.84	3.11	92.39	3.16	39.94	6.14
0.3	99.80	2.66	98.96	2.05	99.41	2.42	90.29	6.22	97.15	4.34	99.60	1.53	97.24	3.71	91.61	4.59
GI50 (ug/ml)	>30		4.843		18.650		1.030		5.558		5.635		6.829		0.080	
GI50 (uM)			11.8		43.8		2.7		13.4		14.8		16.5		0.15	

NCI-H23

Compound	1		2		3		4		5		6		7		8	
Conc.(ug/ml)	Mean	SD	Mean	SD	Mean	SD	Mean	SD	Mean	SD	Mean	SD	Mean	SD	Mean	SD
30	-24.27	0.95	-19.16	7.41	-25.34	0.75	-21.94	3.89	-24.38	7.35	-17.49	0.10	-2.46	6.34	-17.73	4.08
10	35.36	4.86	-13.07	5.52	11.81	3.34	-7.53	5.33	-17.82	4.32	-7.75	3.34	61.62	2.07	13.47	3.68
3	84.65	5.05	21.91	6.19	86.27	7.48	16.92	2.57	31.12	6.27	25.90	4.10	68.23	8.21	70.43	6.35
1	89.23	6.55	44.60	2.44	91.62	5.48	69.95	4.99	62.17	2.90	80.09	7.75	75.87	7.74	86.95	4.62
0.3	96.50	6.23	90.64	8.22	97.92	2.68	91.35	1.12	91.28	5.40	95.97	5.85	98.18	4.26	97.24	2.50
GI50 (ug/ml)	7.090		0.966		5.239		1.433		1.429		1.842		7.122		4.218	
GI50 (uM)	19.6		2.9		14.0		4.0		4.4		4.7		16.1		9.8	

Compound	9		10		11		13		14		15		16		Adriamycin	
Conc.(ug/ml)	Mean	SD	Mean	SD	Mean	SD	Mean	SD	Mean	SD	Mean	SD	Mean	SD	Mean	SD
30	78.92	7.91	22.07	4.69	25.00	6.08	-24.33	3.45	-6.39	1.97	-6.37	6.63	-4.54	7.51	-14.67	1.91
10	88.06	6.93	75.18	3.85	78.50	8.62	-15.73	7.46	31.54	7.20	23.31	6.46	31.79	3.19	-5.25	3.96
3	90.02	5.29	81.71	2.82	89.06	8.73	28.18	9.17	69.68	1.95	76.85	8.77	72.69	6.36	18.10	4.43
1	93.39	4.10	86.18	3.80	92.17	4.91	62.13	5.72	78.28	5.99	88.73	4.83	80.95	4.49	20.39	1.00
0.3	100.52	1.95	94.57	8.61	97.42	6.41	94.49	7.40	93.00	6.73	93.97	5.31	93.59	5.66	93.40	6.96
GI50 (ug/ml)	>30		4.867		17.790		1.412		4.657		5.249		5.125		0.066	
GI50 (uM)			11.9		41.8		3.7		11.2		13.8		12.4		0.12	

PC-3

Compound	1		2		3		4		5		6		7		8	
Conc.(ug/ml)	Mean	SD	Mean	SD	Mean	SD	Mean	SD	Mean	SD	Mean	SD	Mean	SD	Mean	SD
30	-15.01	5.75	-23.54	4.42	-17.29	4.07	-18.15	6.40	-25.37	8.49	-16.09	5.40	-0.50	2.99	-24.43	5.69
10	28.40	4.99	-15.52	6.14	21.65	4.79	-11.73	5.46	-15.33	3.48	-1.03	6.47	71.26	3.26	19.23	4.63
3	79.55	5.57	16.33	4.56	79.71	4.75	27.28	5.82	30.28	4.82	21.12	8.45	89.46	5.29	55.47	5.57
1	91.61	5.24	40.75	5.67	94.51	3.73	58.89	4.20	61.23	9.09	86.83	7.34	96.72	3.46	83.03	4.74
0.3	96.52	4.69	92.69	5.61	99.45	2.32	96.32	6.58	94.38	3.40	98.08	2.64	98.06	4.04	91.59	5.09
GI50 (ug/ml)	5.836		0.883		5.403		1.346		1.422		1.907		11.78		3.232	
GI50 (uM)	16.1		2.7		14.4		3.7		4.4		4.8		26.6		7.5	

Compound	9		10		11		13		14		15		16		Adriamycin	
Conc.(ug/ml)	Mean	SD	Mean	SD	Mean	SD	Mean	SD	Mean	SD	Mean	SD	Mean	SD	Mean	SD
30	68.71	7.66	10.68	0.95	15.81	4.61	-18.37	1.80	-12.38	4.59	-4.10	9.64	-4.33	4.74	-11.55	4.43
10	82.17	6.02	71.33	6.75	70.98	4.23	-16.46	5.07	19.98	6.37	22.79	6.86	15.25	3.96	-4.51	7.69
3	84.81	4.24	82.75	8.75	82.70	4.30	23.03	7.86	72.62	5.08	89.10	9.05	89.51	2.63	18.60	3.85
1	93.23	2.15	94.53	3.60	92.88	4.19	54.04	4.61	96.97	1.49	97.60	3.90	91.48	3.98	31.63	5.14
0.3	96.07	3.39	99.40	1.48	98.54	2.21	96.61	2.54	99.83	2.93	99.15	4.49	97.03	2.63	97.44	2.59
GI50 (ug/ml)	>30		4.182		13.970		1.182		4.802		6.408		5.813		0.079	
GI50 (uM			10.2		32.8		3.1		11.5		16.9		14.0		0.15	

NUGC-3

Compound	1		2		3		4		5		6		7		8	
Conc.(ug/ml)	Mean	SD	Mean	SD	Mean	SD	Mean	SD	Mean	SD	Mean	SD	Mean	SD	Mean	SD
30	-26.66	3.44	-26.53	3.48	-30.32	9.02	-21.59	5.96	-26.17	4.67	-22.84	5.91	-11.29	4.96	-25.05	2.22
10	21.72	4.20	-19.99	3.94	8.93	7.03	-18.51	4.96	-22.93	5.77	-10.86	3.65	65.84	4.98	10.12	6.13
3	44.27	3.84	15.26	4.44	52.41	4.60	21.51	3.46	21.37	4.04	18.83	4.04	70.56	5.65	45.25	4.43
1	86.95	9.33	42.38	4.17	92.58	3.73	61.29	3.30	51.10	7.87	72.34	5.77	82.39	9.12	62.87	6.80
0.3	97.62	3.90	85.49	2.31	99.54	1.96	82.78	7.28	96.23	6.52	94.06	4.82	93.76	1.90	88.45	4.85
GI50 (ug/ml)	2.816		0.849		3.115		1.218		1.098		1.515		8.387		1.860	
GI50 (uM)	7.8		2.6		8.3		3.4		3.4		3.8		18.9		4.3	

Compound	9		10		11		13		14		15		16		Adriamycin	
Conc.(ug/ml)	Mean	SD	Mean	SD	Mean	SD	Mean	SD	Mean	SD	Mean	SD	Mean	SD	Mean	SD
30	70.92	3.98	4.91	6.68	25.36	6.62	-24.75	1.78	-18.41	5.93	-5.68	4.83	-9.34	5.62	-14.47	4.70
10	78.70	4.44	62.75	7.65	60.17	1.77	-15.65	5.61	23.37	9.73	18.80	8.81	20.76	7.41	-1.84	2.43
3	91.00	8.43	76.02	9.03	71.90	5.95	8.75	3.96	67.41	6.49	80.45	6.05	81.55	6.07	19.25	8.08
1	97.88	7.24	88.82	2.34	86.34	7.53	35.44	3.07	85.51	9.80	87.29	4.59	89.09	4.08	32.69	7.46
0.3	98.59	7.06	100.94	6.00	93.37	2.74	83.08	5.55	96.54	4.53	95.74	3.21	91.50	7.66	92.28	3.69
GI50 (ug/ml)	>30		3.147		11.220		0.721		4.314		5.267		5.477		0.079	
GI50 (uM)			7.7		26.3		1.9		10.4		13.9		13.2		0.15	

MDA-MB-231

Compound	1		2		3		4		5		6		7		8	
Conc.(ug/ml)	Mean	SD	Mean	SD	Mean	SD	Mean	SD	Mean	SD	Mean	SD	Mean	SD	Mean	SD
30	-15.61	5.11	-20.08	3.30	-18.47	1.48	-12.47	8.29	-19.15	3.73	-14.46	4.48	-7.18	4.85	-12.12	2.20
10	32.96	3.80	-7.84	2.16	22.50	2.83	-2.98	3.91	-11.98	2.02	2.34	4.67	65.49	6.61	30.13	5.23
3	79.43	6.79	24.33	5.43	83.13	3.67	23.29	8.61	28.32	5.71	30.19	2.71	78.42	7.82	77.56	8.42
1	87.89	3.22	46.31	3.13	96.93	5.47	75.66	2.86	82.84	4.29	89.23	3.60	87.90	5.35	89.16	5.19
0.3	99.67	3.55	91.44	5.84	98.81	4.32	84.88	5.40	93.63	3.44	94.54	2.41	99.02	2.26	91.92	7.52
GI50	6.113		1.029		5.783		1.644		1.962		2.189		10.78		5.723	
GI50 (uM)	16.9		3.1		15.5		4.6		6.0		5.5		24.3		13.3	

Compound	9		10		11		13		14		15		16		Adriamycin	
Conc.(ug/ml)	Mean	SD	Mean	SD	Mean	SD	Mean	SD	Mean	SD	Mean	SD	Mean	SD	Mean	SD
30	75.31	9.25	12.02	8.82	25.96	2.88	-16.03	4.00	-3.44	6.51	-3.99	5.65	-4.34	2.32	-17.81	4.45
10	81.10	4.02	65.60	3.69	58.00	2.41	-12.43	5.90	17.60	8.99	25.88	9.19	27.15	5.72	-6.17	5.64
3	90.00	7.38	72.70	6.56	76.80	5.98	22.16	4.67	82.75	6.56	74.52	1.27	82.93	2.70	23.80	4.79
1	93.10	3.89	83.53	4.33	84.11	4.92	65.75	7.00	91.73	4.72	80.37	8.59	85.44	3.52	36.38	6.20
0.3	93.48	2.24	94.23	5.06	97.11	5.45	98.08	4.94	98.19	4.04	91.42	9.57	99.48	5.08	91.69	3.97
GI50 (ug/ml)	>30		3.188		11.450		1.431		5.428		4.920		6.027		0.086	
GI50 (uM)			7.8		26.9		3.7		13.0		12.9		14.6		0.16	

HCT-15

Compound	1		2		3		4		5		6		7		8	
Conc.(ug/ml)	Mean	SD	Mean	SD	Mean	SD	Mean	SD	Mean	SD	Mean	SD	Mean	SD	Mean	SD
30	-20.73	4.91	-12.67	4.74	-16.10	3.40	-12.69	3.58	-20.52	2.57	-15.53	4.54	-7.22	7.54	-7.06	5.05
10	23.40	7.88	-6.13	2.71	9.59	6.30	-4.30	3.87	-12.43	3.05	-4.11	2.02	67.10	2.47	31.13	5.94
3	72.69	8.67	25.47	10.34	80.13	6.27	20.70	4.51	32.20	3.89	22.41	6.69	78.00	5.55	67.12	5.51
1	86.66	4.07	43.17	7.44	91.28	8.18	66.67	6.39	81.75	6.86	90.76	5.07	88.47	5.70	80.65	4.50
0.3	99.75	4.12	91.78	4.10	97.14	5.01	90.73	3.58	95.03	2.66	95.32	4.94	92.83	3.21	92.15	4.91
GI50 (ug/ml)	4.779		0.993		4.691		1.414		2.037		2.035		10.900		4.508	
GI50 (uM)	13.2		3.0		12.5		3.9		6.2		5.2		24.6		10.5	

Compound	9		10		11		13		14		15		16		Adriamycin	
Conc.(ug/ml)	Mean	SD	Mean	SD	Mean	SD	Mean	SD	Mean	SD	Mean	SD	Mean	SD	Mean	SD
30	65.62	7.58	7.25	7.55	28.84	5.33	-10.59	1.32	-4.27	4.23	-1.17	4.63	1.45	3.58	-13.54	2.53
10	78.29	8.68	64.56	5.58	83.31	2.86	-4.38	1.82	19.16	6.99	25.74	7.38	31.03	4.07	-9.76	6.71
3	81.38	9.03	87.21	2.35	90.77	3.17	27.63	6.64	81.69	3.88	78.10	2.50	64.00	5.83	12.95	8.63
1	90.04	6.95	89.85	3.47	94.41	5.23	79.33	4.22	89.27	0.81	84.40	4.59	79.24	7.16	31.53	4.09
0.3	96.81	1.77	100.41	2.67	96.14	2.18	95.91	4.16	92.81	4.06	92.03	4.36	94.16	5.63	97.28	3.80
GI50 (ug/ml)	>30		3.718		19.970		1.869		5.417		5.438		4.354		0.078	
GI50 (uM)			9.1		46.9		4.9		13.0		14.3		10.5		0.15	

Figure S31. Results of the cytotoxicity test of compounds **1-11** and **13-16**.
